# The Wnt5a-Ror2 axis promotes the signaling circuit between interleukin-12 and interferon-γ in colitis

**DOI:** 10.1038/srep10536

**Published:** 2015-06-01

**Authors:** Akira Sato, Hisako Kayama, Kensaku Shojima, Shinji Matsumoto, Hirofumi Koyama, Yasuhiro Minami, Satoshi Nojima, Eiichi Morii, Hiroaki Honda, Kiyoshi Takeda, Akira Kikuchi

**Affiliations:** 1 Departments of Molecular Biology and Biochemistry; 2 Microbiology and Immunology, and; 3 Pathology, Graduate School of Medicine, Osaka University, 2-2 Yamadaoka, Suita 565-0871, Japan; 4 Department of Disease Model, Research Institute of Radiation Biology and Medicine, Hiroshima University, 1-2-3 Kasumi, Minami-ku, Hiroshima 734-8553, Japan; 5 Department of Physiology and Cell Biology, Graduate School of Medicine, Kobe University, 7-5-1 Kusunoki-cho, Chuoh-ku, Kobe 650-0017, Japan

## Abstract

Wnt5a, which regulates various cellular functions in Wnt signaling, is involved in inflammatory responses, however the mechanism is not well understood. We examined the role of Wnt5a signaling in intestinal immunity using conditional knockout mice for Wnt5a and its receptor Ror2. Removing Wnt5a or Ror2 in adult mice suppressed dextran sodium sulfate (DSS)-induced colitis. It also attenuated the DSS-dependent increase in inflammatory cytokine production and decreased interferon-γ (IFN-γ)-producing CD4^+^ Th_1_ cell numbers in the colon. Wnt5a was highly expressed in stromal fibroblasts in ulcerative lesions in the DSS-treated mice and inflammatory bowel disease patients. Dendritic cells (DCs) isolated from the colon of *Wnt5a* and *Ror2* deficient mice reduced the ability to differentiate naïve CD4^+^ T cells to IFN-γ-producing CD4^+^ Th_1_ cells. *In vitro* experiments demonstrated that the Wnt5a-Ror2 signaling axis augmented the DCs priming effect of IFN-γ, leading to enhanced lipopolysaccharide (LPS)-induced interleukin (IL)-12 expression. Taken together, these results suggest that Wnt5a promotes IFN-γ signaling, leading to IL-12 expression in DCs, and thereby inducing Th_1_ differentiation in colitis.

Wnt5a is a representative ligand that activates the Wnt/β-catenin independent signaling pathway and is one of the most extensively studied ligand in the Wnt family. It plays important roles in the developmental processes of various organs and has postnatal cellular functions^1^,^2^. Wnt5a binds to and internalizes its receptor complex, which consists of Frizzled (Fz), a seven transmembrane receptor, and receptor tyrosine kinase-like orphan receptor (Ror) 1 or Ror2, a single transmembrane receptor. Wnt5a binding activates Rho, Rac, protein kinase C, and Jun-N-terminal kinase (JNK), thereby regulating the cytoskeleton, cell migration and polarity, and gene expression^3^,^4^.

Mice homozygous for a *Wnt5a* null allele (*Wnt5a*^*−/−*^) suffer perinatal lethality because of asphyxia^5^. The embryos are truncated caudally, displaying an inability to extend the embryonic anterior-posterior axis. Furthermore, knockout of *Wnt5a* down-regulates expression of the pro-apoptotic gene *Bax*, and promotes expression of the anti-apoptotic gene *Bcl-2,* which inhibits apoptosis in CD4^+^CD8^+^ double positive thymocytes, suggesting that Wnt5a plays a role in hematopoietic cell development^6^,^7^. Although there are no gross abnormalities in the post-natal development of *Wnt5a* heterozygous (*Wnt5a*^*+/−*^) mice, they have a bone-loss phenotype with decreased trabecular bone mass^8^. Aged *Wnt5a*^*+/−*^ mice have an increased population of B cells and develop myeloid and B-cell leukemia^9^. In addition, it has been suggested that post-natal abnormalities in Wnt5a signaling are involved in inflammatory diseases, as well as cancers^1^.

For instance, expression of Wnt5a and Fz5 can be induced through Toll-like receptors (TLR) stimulated by *Mycobacterium tuberculosis* (*M. tuberculosis*) or lipopolysaccharide (LPS) in macrophages, and Wnt5a signaling is involved in producing pro-inflammatory cytokines, such as interleukin (IL)-12 and IL-6^10^,^11^,^12^. Wnt5a has been detected in granulomatous lesions in the lungs of patients with *M. tuberculosis*, bone marrow macrophages in septic patients, and macrophages accumulated within the intima of atherosclerotic patients^10^,^11^,^13^. Thus, these results suggest that Wnt5a released from macrophages in response to inflammatory cues affects macrophages in an autocrine manner to release cytokines. However, the underlying mechanism by which Wnt5a regulates inflammation remains enigmatic. Although purified Wnts are useful tools to better understand the roles of Wnt signaling in the inflammatory responses, it has been reported that recombinant Wnt preparations contain TLR agonists that may lead to inflammatory cytokine production^14^. Therefore, further *in vivo* studies elucidating the roles of Wnt5a signaling in the immune responses using adult *Wnt5a* knockout mice are necessary.

Interferon-γ (IFN-γ) is a key immunoregulatory protein that plays a major role in the host innate and adaptive immune responses^15^. IFN-γ is mainly produced in Th_1_ cells, which are differentiated from naïve T cells by IL-12 released from antigen-presenting cells, including dendritic cells (DCs) and macrophages^15^,^16^. Engagement of IFN-γ with its receptor leads to the activation of Janus kinase (JAK) and the phosphorylation of signal transducer and activator of transcription (STAT)-1^17^,^18^. STAT1 then translocates into the nucleus where it binds to DNA and initiates the transcription of the STAT1 target genes. IFN-γ also has a priming function and increases inflammatory cytokine production, including tumor necrosis factor-α (TNF-α), IL-6, and IL-12, in response to TLR ligands in DCs and macrophages^15^,^16^. Thus, it appears that IFN-γ and IL-12 form a signaling circuit between Th_1_ cells and antigen-presenting cells.

Here we use dextran sodium sulfate (DSS)-induced colitis in mice as a model for inflammatory diseases and show that disease symptoms were milder in *Wnt5a* and *Ror2* conditional knockout mice than control mice. Blocking Wnt5a signaling also reduced the production of pro-inflammatory cytokines in the colon. Finally, we demonstrate that the Wnt5a-Ror2 axis enhances the priming action of IFN-γ to increase TLR-dependent production of IL-12 in DCs, thereby promoting immune responses.

## Results

### Wnt5a knockout mice were less susceptible to DSS-induced colitis

**Figure 1:**
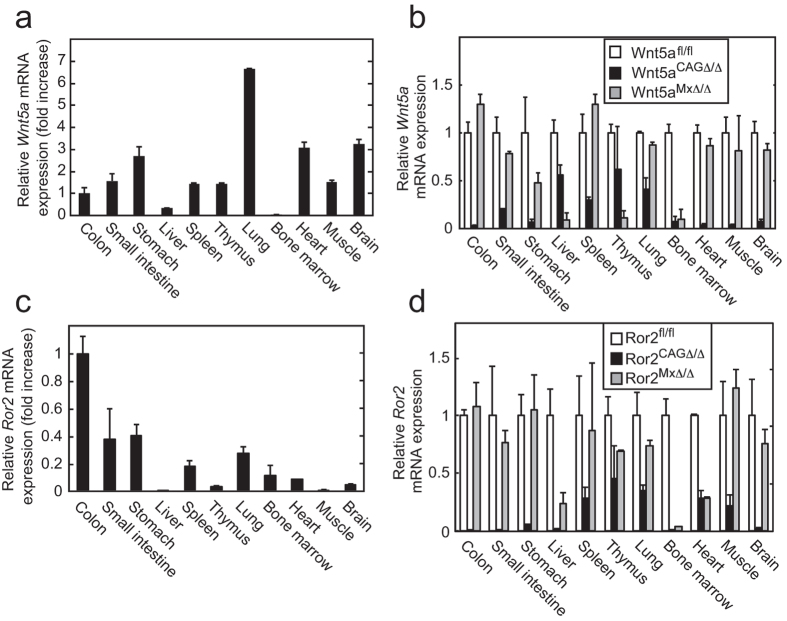
The *Wnt5a* and *Ror2* mRNA levels in various tissues. (**a**) The *Wnt5a* mRNA levels in various organs isolated from control *Wnt5a*^*fl/fl*^ mice (n = 3) were measured using quantitative RT-PCR. Expression level of each tissue was normalized to that of colon. (**b**) The *Wnt5a* mRNA levels in the indicated organs isolated from *Wnt5a*^*CAGΔ/Δ*^ (n = 3) and *Wnt5a*^*MxΔ/Δ*^ mice (n = 3) were expressed compared with expression levels from *Wnt5a*^*fl/fl*^ mice (n = 3). (**c**) The *Ror2* mRNA levels in various organs isolated from control *Ror2*^*fl/fl*^ mice (n = 3). Expression level of each tissue was normalized to that of colon. (**d**) The *Ror2* mRNA levels in the indicated organs isolated from *Ror2*^*CAGΔ/Δ*^ (n = 3) and *Ror2*^*MxΔ/Δ*^ (n = 5) mice were expressed compared with expression levels from *Ror2*^*fl/fl*^ mice (n = 3). The results are shown as means ± SE.

Given that *Wnt5a*^*−/−*^ mice suffer perinatal lethality^5^, we generated *Wnt5a*^*flox/flox*^ (*Wnt5a*^*fl/fl*^) mice (Figures S1A-C), in which exon 2 of the *Wnt5a* gene was flanked by loxP sites. These mice were crossed with different Cre-expressing mice, including *CAG-Cre/ERT2*^*Tg*^ mice, and the offspring were treated with DSS. *CAG-Cre/ERT2*^*Tg*^ mice show ubiquitous expression of Cre/ERT2 which is activated by administration of tamoxifen^19^. In the *Wnt5a*^*fl/fl*^*;CAG-Cre/ERT2*^*Tg*^ mice, *Wnt5a* exon2 was deleted in the colon by administering tamoxifen (*Wnt5a*^*CAGΔ/Δ*^ mice) (Figure S1D). *Wnt5a* mRNA expression levels varied between *Wnt5a*^*fl/fl*^ mouse tissues, but it was remarkably lower in the liver and bone marrow than other tissues (Fig. 1a). In *Wnt5a*^*CAGΔ/Δ*^mice, *Wnt5a* mRNA was dramatically decreased in the colon, small intestine, stomach, bone marrow, heart, muscle, and brain; and expression was reduced by half in the liver, spleen, thymus, and lung (Fig. 1b).

**Figure 2:**
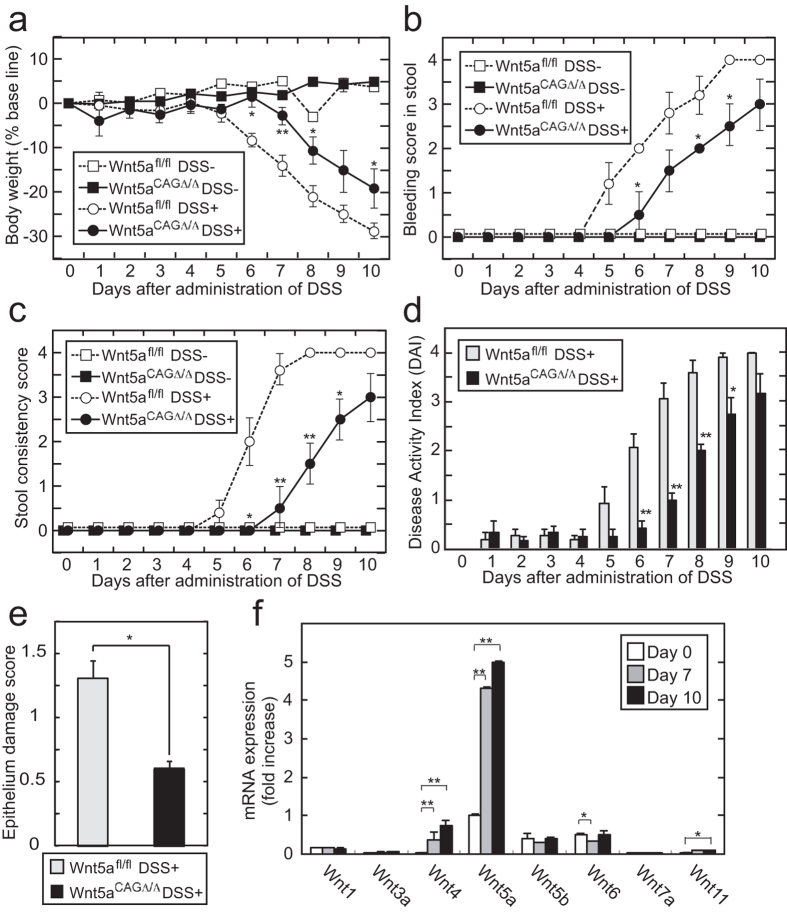
*Wnt5a*^*CAGΔ/Δ*^mice show resistance to DSS-induced colitis. (**a**–**c**) *Wnt5a*^*fl/fl*^ (n = 5) and *Wnt5a*^*CAGΔ/Δ*^ mice (n = 5) were given DSS for 10 days. As a control, *Wnt5a*^*fl/fl*^ (n = 3) and *Wnt5a*^*CAGΔ/Δ*^ mice (n= 3) were given water. Percent body weight loss (**a**), bleeding score (**b**), and stool consistency/diarrhea score (**c**) of the mice were measured daily. (**d**) DAI was calculated from the scores of body weight loss, stool consistency, and gross bleeding. (**e**) The epithelium damage was expressed as the average of the scores of the four pathological classifications. An approximately 1 cm length of the colon from the anus was isolated after *Wnt5a*^*fl/fl*^ (n = 3) and *Wnt5a*^*CAGΔ/Δ*^ mice (n = 3) were administrated with DSS for 7 days, and histologically analyzed. Four histological patterns were classified as follows: intact crypts, decreased crypt lesions, monolayer lesions, and ulcer lesions. The histological patterns were expressed as the frequencies of the four pathological classifications (see Figures S2A–D). (**f**) WT mice (n = 3) were given DSS for the indicated number of days. Total RNA was extracted from a 1 cm length of the colon from the anus, and the mRNA levels of the indicated *Wnts* were expressed as fold increases compared with the *Wnt5a* mRNA level at day 0. The results are shown as means ± SD (**a**–**e**) or SE (**f**). *P < 0.05, **P < 0.01 as calculated by one-way ANOVA (**a**–**e**) and by the Student’s *t*-test (**f**).

DSS (2.5%) was delivered in drinking water to *Wnt5a*^*CAGΔ/Δ*^mice and their *Wnt5a*^*fl/fl*^ littermates. *Wnt5a*^*CAGΔ/Δ*^mice showed less weight loss than the WT mice following colitis induction (Fig. 2a). DSS caused bleeding in the stools (Fig. 2b) and pasty stools (Fig. 2c) at 5–6 days after its administration. These phenotypes were observed later and to a lesser degree in *Wnt5a*^*CAGΔ/Δ*^mice (Fig. 2b,c). The overall severity of colitis measured as the disease activity index (DAI), which included scores for body weight loss, occult and gross stool bleeding, and stool consistency, was milder in *Wnt5a*^*CAGΔ/Δ*^mice than *Wnt5a*^*fl/fl*^ mice (Fig. 2d). These results suggested that *Wnt5a*^*CAGΔ/Δ*^mice were less susceptible to DSS-induced colitis.

The histological patterns in the colon of DSS-fed mice were classified into 4 categories (Figures S2A-D); (1) intact crypts, (2) decreased crypt lesions, (3) monolayer lesions, and (4) ulcer lesions. In WT mice, the frequencies of decreased crypt, monolayer, and ulcer lesions increased gradually over 5 days of DSS administration (Figure S2E). However, the histopathological scores of epithelium damage were reduced in the DSS-fed *Wnt5a*^*CAGΔ/Δ*^ mice compared with *Wnt5a*^*fl/fl*^ mice (Fig. 2e and Figure. S2F). Although several *Wnt* mRNAs, including *Wnt1, Wnt4, Wnt5a, Wnt5b, Wnt6,* and *Wnt11*, were expressed in the colon, *Wnt5a* mRNA was expressed more highly than other *Wnt* mRNAs, and significantly increased by DSS administration (Fig. 2f). Thus, Wnt5a might be involved in DSS-induced colitis.

### Wnt5a was required for inflammatory cytokine production in the colon

**Figure 3:**
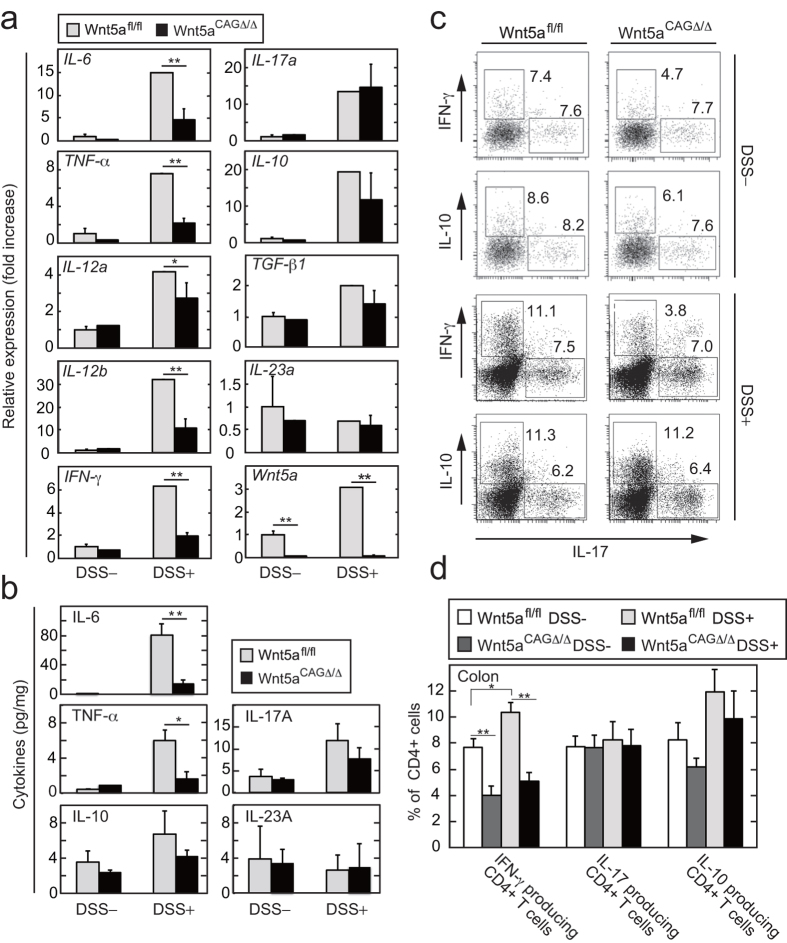
Wnt5a is involved in pro-inflammatory cytokine production in the colon. (**a**) *Wnt5a*^*fl/fl*^ (n = 4) and *Wnt5a*^*CAGΔ/Δ*^ (n = 4) mice were treated with DSS for 6 days. As controls, *Wnt5a*^*fl/fl*^ (n = 3) and *Wnt5a*^*CAGΔ/Δ*^ (n = 3) mice were untreated. Total RNA was extracted from a 1 cm length of the colon from the anus. The mRNA levels of the indicated cytokines were measured by quantitative RT-PCR and expressed as fold increases compared with control *Wnt5a*^*fl/fl*^ mice. (**b**) *Wnt5a*^*fl/fl*^ (n = 5) and *Wnt5a*^*CAGΔ/Δ*^(n = 3) mice were treated with DSS for 7 days. As controls, *Wnt5a*^*fl/fl*^ (n = 3) and *Wnt5a*^*CAGΔ/Δ*^ (n = 3) mice were untreated. The supernatants of the lysates of the colon were prepared and the amounts of the indicated cytokines were measured by ELISA. (**c**) Representative FACS plots showing the percentage of colon CD4^+^ T cells that produce IFN-γ, IL-17, and IL-10 in the colon isolated from control *Wnt5a*^*fl/fl*^ (n = 5) and *Wnt5a*^*CAGΔ/Δ*^ (n = 4) mice or DSS-administrated *Wnt5a*^*fl/fl*^ (n = 6) and *Wnt5a*^*CAGΔ/Δ*^ (n = 6) mice. (**d**) The percentage of CD4^+^ T cells that produce IFN-γ, IL-17, and IL-10 in Figure 3c were expressed as means ± SD. The results are shown as means ± SE (**a** and **b**) or SD (**d**). *P < 0.05, **P < 0.01 as calculated by the Student’s *t*-test (**a** and **b**) and by one-way ANOVA (**d**).

Many ulcer lesions were observed in the area within a 1 cm distance from the anus in DSS-induced colitis. In the lesions of *Wnt5a*^*fl/fl*^ mice, DSS treatment induced increased mRNA expression levels of *IL-6*, *TNF-*α, *IL-12a*, *IL-12b*, and *IFN-*γ (Fig. 3a). The increases were suppressed in the colon of *Wnt5a*^*CAGΔ/Δ*^ mice (Fig. 3a). The mRNA levels of *IL-17a*, *IL-10*, and *transforming growth factor-β1 (TGF-*β1) were increased in the colons of both *Wnt5a*^*fl/fl*^ and *Wnt5a*^*CAGΔ/Δ*^mice by DSS treatment, but their expression levels were not significantly reduced in *Wnt5a*^*CAGΔ/Δ*^ mice (Fig. 3a). The *IL-23a* mRNA level was not changed in the colon of DSS-fed *Wnt5a*^*fl/fl*^ and *Wnt5a*^*CAGΔ/Δ*^ mice (Fig. 3a). Enzyme-linked immunosorbent assay (ELISA) confirmed that DSS-induced production of IL-6 and TNF-α was decreased in the *Wnt5a*^*CAGΔ/Δ*^ mouse colon and that production of IL-10, IL-17A, and IL-23A was not changed in *Wnt5a*^*CAGΔ/Δ*^ and *Wnt5a*^*fl/fl*^ mice (Fig. 3b). These results suggested that DSS-induced pro-inflammatory cytokine synthesis in the colon was decreased in *Wnt5a*^*CAGΔ/Δ*^ mice compared with control *Wnt5a*^*fl/fl*^ mice.

It is thought that intestinal inflammation is caused by an imbalance between the inflammatory response and tolerance^20^,^21^. Therefore, the frequencies of CD4^+^ Th_1_, Th_17_, and T_reg_ cells in various tissues were compared in *Wnt5a*^*fl/fl*^ and *Wnt5a*^*CAGΔ/Δ*^ mice. In the colon, *Wnt5a*^*CAGΔ/Δ*^ mice had reduced frequencies of IFN-γ-producing cells (Th_1_ cells) compared with *Wnt5a*^*fl/fl*^ mice in the absence or presence of DSS treatment (Fig. 3c,d). In contrast, the frequency of IL-10-producing T cells (T_reg_ cells) was slightly, not significantly, decreased, and the frequency of IL-17-producing T cells (Th_17_ cells) was not affected by Wnt5a deletion (Fig. 3c,d). In the small intestine, although the frequency of Th_17_ cells was slightly, not significantly, decreased by Wnt5a deletion, the frequencies of others were not affected (Figure S3A). Loss of *Wnt5a* did not affect the frequencies of Th_1_, Th_17_, and T_reg_ cells in the mesenteric lymph nodes, spleen, and thymus (Figure S3B). These results suggested that Wnt5a deficiency suppresses Th_1_ polarization process, including T cell recruitment and differentiation^22^,^23^, in the colon, thereby attenuating DSS-induced colitis. However, it is unlikely that T cell recruitment from the mesenteric lymph nodes to the colon was decreased in *Wnt5a*^*CAGΔ/Δ*^ mice, because the number of total CD4^+^ T cells in the colon and the mesenteric lymph nodes was not changed between *Wnt5a*^*fl/fl*^ and *Wnt5a*^*CAGΔ/Δ*^mice (Figure S3C). The number of Th_1_ cells was reduced in the colon of *Wnt5a*^*CAGΔ/Δ*^ mice rather than that of *Wnt5a*^*fl/fl*^mice, whereas that of T cells was not increased in the mesenteric lymph nodes (Figure S3C). Therefore, Wnt5a might be involved in Th_1_ differentiation.

### Loss of Wnt5a in the bone marrow and intestinal epithelium did not affect DSS-induced colitis

Given that LPS induces Wnt5a expression in bone marrow cells and macrophages and that Wnt5a alters DC responses to LPS^10^,^11^,^24^, we first speculated that the inhibition of DSS-induced colitis might be caused by the loss of Wnt5a in hematopoietic cells. To address this question, *Wnt5a*^*fl/fl*^*;Mx-Cre*^*Tg*^ mice were generated. Mx-Cre mainly deletes genes in hematopoietic cells^25^. In these mice *Wnt5a* mRNA expression levels were decreased in the liver, thymus, and bone marrow by the peritoneal administration of polyinosine-polycytosine (pIpC) (*Wnt5a*^*MxΔ/Δ*^ mice) (Fig. 1b). Genomic DNA isolated from the bone marrow of *Wnt5a*^*MxΔ/Δ*^ mice showed efficient digestion of *Wnt5a* exon2 compared with that of *Wnt5a*^*fl/fl*^ mice (Figure S4A). However, the *Wnt5a*^*MxΔ/Δ*^ mice had colitis phenotypes that were similar in terms of severity to *Wnt5a*^*fl/fl*^ mice (Figures S4B and C).

Villin-Cre depletes genes in the epithelial cells of the intestines^26^. In *Wnt5a*^*fl/fl*^*;Villin-Cre*^*Tg*^ mice (*Wnt5a*^*VilΔ/Δ*^ mice), *Wnt5a* genomic DNA was digested in the colon (Figure S4D). However, the reduction of *Wnt5a* mRNA in the colon was not clearly observed (Figure S4D), likely because Wnt5a might be mainly expressed in the regions other than epithelium of the colon. The clinicopathological symptoms of DSS-induced colitis in the *Wnt5a*^*VilΔ/Δ*^ mice were unchanged compared with *Wnt5a*^*fl/fl*^ mice (Figures S4E and F). Thus, the Wnt5a, if any, released from hematopoietic and intestinal epithelial cells could not be involved in colitis, and Wnt5a produced in other cells may play roles in colitis.

### Ror2 deficient mice were also less susceptible to DSS-induced colitis

**Figure 4:**
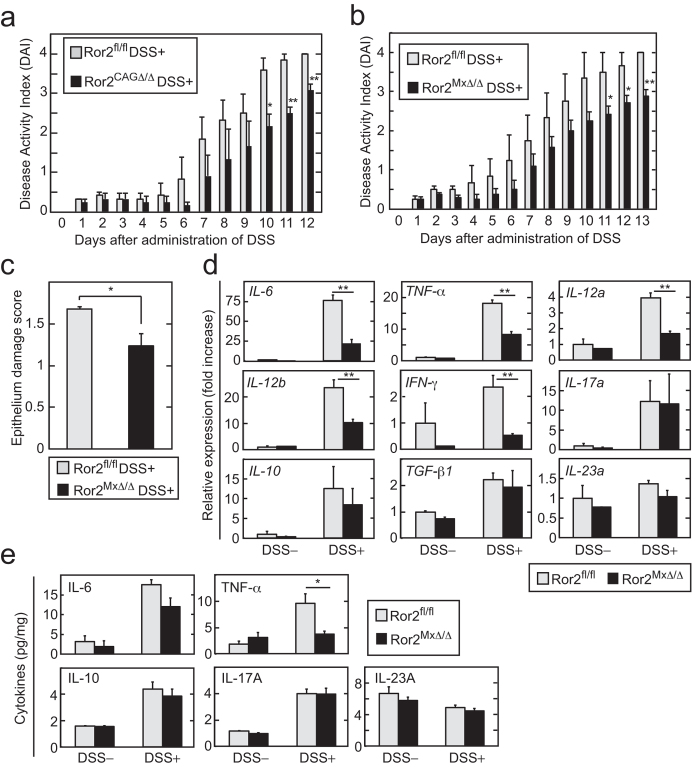
*Ror2*^*MxΔ/Δ*^ mice show resistance to DSS-induced colitis. (**a**) *Ror2*^*fl/fl*^ (n = 5) and *Ror2*^*CAGΔ/Δ*^ mice (n = 8) were given DSS for 12 days. The DAI was calculated daily. (**b**) *Ror2*^*fl/fl*^ (n = 4) and *Ror2*^*MxΔ/Δ*^ mice (n = 8) were given DSS for 13 days. The DAI was calculated daily. (**c**) The epithelium damage score of *Ror2*^*fl/fl*^ (n = 3) and *Ror2*^*MxΔ/Δ*^ mice (n = 3) administrated with DSS for 7 days was calculated. (**d**) DSS was administered to *Ror2*^*fl/fl*^ (n = 7) and *Ror2*^*MxΔ/Δ*^ (n = 5) mice for 7 days. As control, *Ror2*^*fl/fl*^ (n = 3) and *Ror2*^*MxΔ/Δ*^(n = 3) mice were untreated. The mRNA levels of the indicated cytokines were measured by quantitative RT-PCR and expressed as fold increases compared with control *Ror2*^*fl/fl*^ mice. (**e**) DSS was administered to *Ror2*^*fl/fl*^ (n = 3) and *Ror2*^*MxΔ/Δ*^ (n = 3) mice for 7 days. As control, *Ror2*^*fl/fl*^ (n = 3) and *Ror2*^*MxΔ/Δ*^(n = 3) mice were untreated. The supernatants of the lysates of the colon were prepared and the amounts of the indicated cytokines were measured by ELISA. The results are shown as means ± SD (**a**–**c**) or SE (**d** and **e**). *P < 0.05, **P < 0.01 as calculated by one-way ANOVA (**a**–**c**), and the Student’s *t*-test (**d** and **e**).

Ror2 is one of the receptors for Wnt5a. *Ror2*^*−/−*^ mice are embryonic lethal and display phenotypes similar to *Wnt5a*^*−/−*^ mice^27^. To examine whether Ror2 is involved in DSS-induced colitis, *Ror2*^*fl/fl*^*;CAG-Cre/ERT2*^*Tg*^ and *Ror2*^*fl/fl*^*;Mx-Cre^Tg^* mice were administrated tamoxifen (*Ror2*^*CAGΔ/Δ*^) and pIpC (*Ror2*^*MxΔ/Δ*^), respectively, and then DSS was administrated to these mice. *Ror2* mRNA was variably expressed in *Ror2*^*fl/fl*^ mouse tissues, but the liver, thymus, and muscle had the lowest expression levels (Fig. 1c). *Ror2* mRNA expression was reduced in most tissues of *Ror2*^*CAGΔ/Δ*^ mice, and especially in the liver, bone marrow, and heart of the *Ror2*^*MxΔ/Δ*^ mice (Fig. 1d). Compared with the *Ror2*^*fl/fl*^ mice, both types of *Ror2* conditional knockout mice exhibited milder signs and pathological changes following DSS-induced colitis (Fig. 4a,b). The histopathological scores of epithelium damage were also reduced in the DSS-fed *Ror2*^*MxΔ/Δ*^mice compared with *Ror2*^*fl/fl*^ mice (Fig. 4c and Figure. S2G). It is noteworthy that *Ror2*^*MxΔ/Δ*^ mice suppressed DSS-induced colitis in contrast to *Wnt5a*^*MxΔ/Δ*^ mice. The production of pro-inflammatory cytokines, including IL-6 and TNF-α, was suppressed in the colon of *Ror2*^*MxΔ/Δ*^ mice, similar to the *Wnt5a*^*CAGΔ/Δ*^ mice (Fig. 4d,e). Taken together, these results suggested that Ror2 in hematopoietic cells was involved in progression of DSS-induced colitis.

### Wnt5a was released primarily from fibroblasts and Ror2 was expressed in DCs in the colon

**Figure 5:**
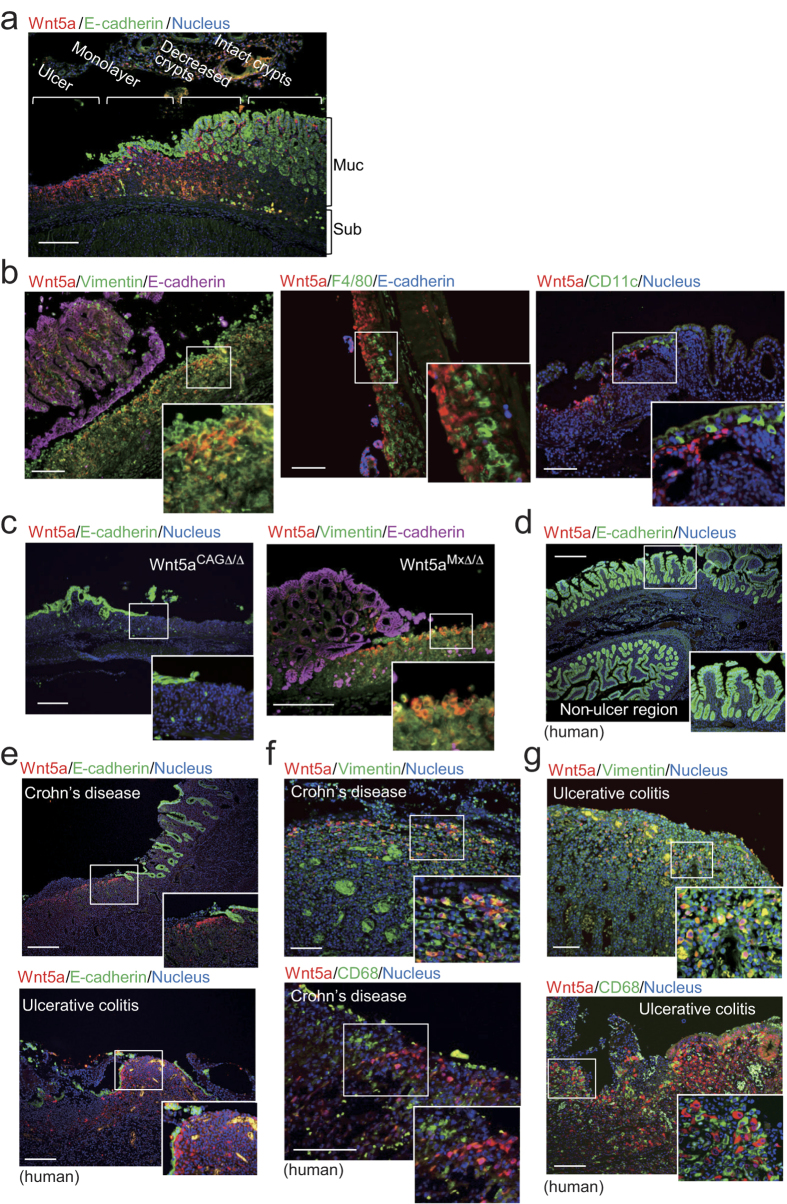
DSS causes Wnt5a expression primarily in fibroblasts. (**a** and **b**) Tissue sections of the colon at day 10 after administration of DSS were stained with indicated antibodies and DRAQ5. Scale bars, 200 μm (**a**); 100 μm (**b**). (**c**) Tissue sections of the colon isolated from *Wnt5a*^*CAGΔ/Δ*^ (*left panel*) and *Wnt5a*^*MxΔ/Δ*^(*right panel*) mice at day 10 after administration of DSS were stained with indicated antibodies and DRAQ5. Scale bars, 200 μm. (**d**) A tissue section of the intact crypt regions in the human colon was stained with indicated antibodies and DRAQ5. Scale bars, 200 μm. (**e**) Tissue sections of the colon isolated from Crohn’s disease (*top panel*) and ulcerative colitis (*bottom panel*) patients were stained with indicated antibodies and DRAQ5. Scale bars, 200 μm. (**f** and **g**) Tissue sections of the colon isolated from Crohn’s disease (**f**) and ulcerative colitis (**g**) patients were stained with indicated antibodies and DRAQ5. Scale bars, 100 μm.

To examine which cells are the major source of Wnt5a, the colons from mice fed with DSS were analyzed for Wnt5a expression. *Wnt5a* mRNA was expressed in fibroblasts more highly than epithelial cells and hematopoietic cells, including CD4^+^ T cells, B220^+^ B cells, CD11c^+^ DCs, CD11b^+^CD11c^−^ macrophages, and γδT cells in the colon, and its expression level was elevated only in the fibroblasts after DSS administration (Figure S5A). In DSS-fed mice, Wnt5a protein and *Wnt5a* mRNA were not detected in the intact crypt regions of the colon, whereas the protein and mRNA were clearly visible in the mesenchyme of the ulcer lesions (Fig. 5a and Figure. S6). Wnt5a-expressing cells were positive for vimentin (a mesenchymal cell marker), but not for F4/80 (a macrophage marker) and CD11c (a DC marker) (Fig. 5b), suggesting that they were fibroblasts. In addition, Wnt5a was still expressed in the mesenchyme of ulcer lesions in *Wnt5a*^*MxΔ/Δ*^ mice but not in *Wnt5a*^*CAGΔ/Δ*^ mice (Fig. 5c). Taken together, these results suggested that Wnt5a secreted from cells other than hematopoietic cells, probably fibroblasts, affects DSS-induced colitis.

Wnt5a expression was then examined in the colon from Crohn’s disease (9 cases) and ulcerative colitis (10 cases) patients. Wnt5a was not detected in the intact crypt regions (E-cadherin positive) (Fig. 5d), but it was clearly detected in the mesenchyme of ulcer lesions (E-cadherin negative) in about half of the inflammatory bowel disease cases (Fig. 5e). As in the mice, the Wnt5a was detected in vimentin-positive cells but not in the cells expressing CD68, a macrophage and monocyte marker (Fig. 5f,g).

In contrast to Wnt5a, *Ror2* mRNA was expressed in hematopoietic cells although it was lower than in fibroblasts (Figure S5B). Among hematopoietic cells, DCs as well as B cells, macrophages, and γδT cells, showed higher *Ror2* mRNA expression compared with T cells. However, DSS treatment reduced *Ror2* mRNA expression in B and γδT cells (Figure S5B), suggesting that these cells are hard to respond to Wnt5a in DSS-induced colitis. In addition, cell numbers of γδT cells were not changed between *Wnt5a*^*CAGΔ/Δ*^, *Ror2*^*MxΔ/Δ*^, and their control mice irrespective of the presence or the absence of DSS treatment (Figure S7). Taken together with the observations that γδT cells play a role in protections against DSS-induced colitis^28^, DCs might respond to Wnt5a in colitis rather than B, T, and γδT cells.

Intestinal DC subsets have been identified by the combination and expression levels of specific cell surface antigens and characterized by their functions in immune responses^29^,^30^. We classified CD11c^+^ DCs into three subsets, including CD11c^+^CD11b^−^CD103^+^ cells that induce Th_1_ polarization, CD11c^+^CD11b^+^CD70^+^CX_3_CR1^intermediate^ cells that induce Th_17_ polarization, and CD11c^+^CD11b^+^ CD70^+^CX_3_CR1^high^ cells that inhibit T cell proliferation^29^,^30^,^31^,^32^. The *Ror2* mRNA levels varied among subsets and CD11c^+^CD11b^−^CD103^+^ DC cells indeed expressed *Ror2* mRNA (Figure S5C), and loss of *Wnt5a* or *Ror2* did not affect total cell numbers of DC subsets in the presence or the absence of DSS treatment (Figure S7). These results suggest that Wnt5a-Ror2 signaling is involved in the activation of intestinal DCs expressing Ror2 rather than their differentiation.

### The Wnt5a-Ror2 axis was involved in pro-inflammatory cytokine synthesis in DCs

**Figure 6:**
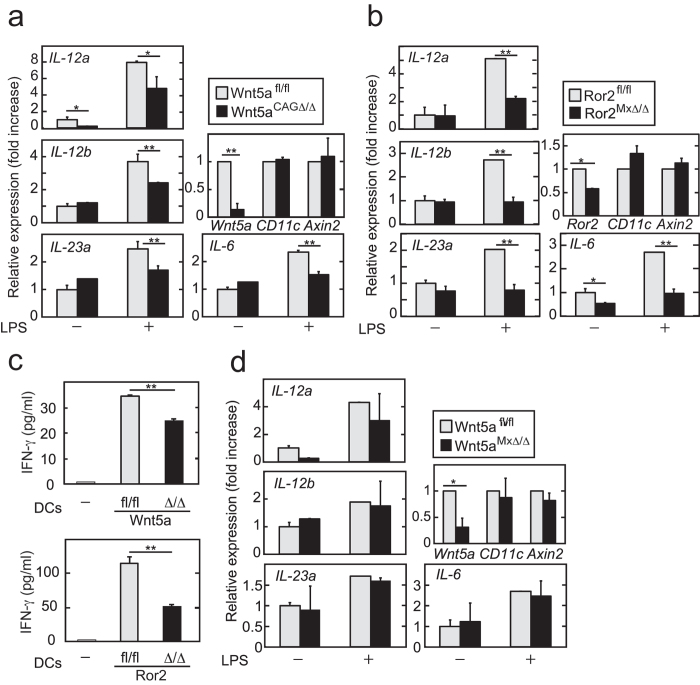
The Wnt5a-Ror2 axis is involved in pro-inflammatory cytokine synthesis in CD11c^+^DCs. (**a** and **b**) CD11c^+^ DCs isolated from the colon of *Wnt5a*^*fl/fl*^ or *Wnt5a*^*CAGΔ/Δ*^ mice (**a**) and *Ror2*^*fl/fl*^ or *Ror2*^*MxΔ/Δ*^mice (**b**) were stimulated with 100 ng/ml LPS for 4 h and the total RNA was extracted. The mRNA levels of the indicated genes were measured by quantitative RT-PCR and expressed as fold increases compared with expression in control CD11c^+^ DCs without LPS stimulation. (**c**) Splenic naïve CD4^+^ T cells were cocultured with CD11c^+^ DCs isolated from the colon of *Wnt5a*^*fl/fl*^ (n = 3) or *Wnt5a*^*CAGΔ/Δ*^ (n = 3) mice (*top panel*), or *Ror2*^*fl/fl*^ (n = 3) or *Ror2*^*MxΔ/Δ*^ (n = 3) mice (*bottom panel*) in the presence of 1 μg/ml anti-CD3 antibody for 24 h. The concentrations of IFN-γ in the culture supernatants were measured by ELISA. The results were shown as means ± SD. (**d**) CD11c^+^ DCs isolated from the colon of control *Wnt5a*^*fl/fl*^ or *Wnt5a*^*MxΔ/Δ*^ mice were stimulated with 100 ng/ml LPS for 4 h. The mRNA levels of the indicated genes were measured by quantitative RT-PCR and expressed as fold increases compared with expression in control CD11c^+^ DCs without LPS stimulation. Two indicated conditional knockout mice were used in one experiment (**a**,**b** and **d**). The results are shown as means ± SE from three independent experiments (**a**,**b** and **d**). *P < 0.05, **P < 0.01 as calculated by the Student’s *t*-test (**a**,**b** and **d**) and by one-way ANOVA (**c**).

Intestinal DCs and macrophages play important roles in the regulation of gut homeostasis through induction of helper T cell subsets^20^,^21^. Therefore, intracellular signaling cascades for cytokine productions involved in Th_1_ differentiation were examined in DCs. LPS induced the expression of *IL-12a*, *IL-12b*, *IL-23a*, and *IL-6* mRNA in CD11c^+^ DCs from *Wnt5a*^*fl/fl*^ and *Ror2*^*fl/fl*^ mice (Fig. 6a,b). However, LPS-induced increases in the expression of these cytokines were reduced in DCs from *Wnt5a*^*CAGΔ/Δ*^ and *Ror2*^*MxΔ/Δ*^ mice (Fig. 6a,b). Colon CD11c^+^ DCs induces Th_1_ differentiation from naïve T cells *in vitro*^33^,^34^. The ability of DCs from *Wnt5a*^*CAGΔ/Δ*^ and *Ror2*^*MxΔ/Δ*^ mice to induce Th_1_ differentiation was decreased compared with those from *Wnt5a*^*fl/fl*^ and *Ror2*^*fl/fl*^ mice (Fig. 6c), suggesting that Wnt5a signaling through Ror2 in CD11c^+^ DCs is required for cytokine expression which induces Th_1_ differentiation. It is noteworthy that LPS-induced production of *IL-12a*, *IL-12b*, *IL-23a*, and *IL-6* mRNA was not suppressed in colon CD11c^+^ DCs from *Wnt5a*^*MxΔ/Δ*^ mice (Fig. 6d), which are consistent with the observation that *Wnt5a*^*MxΔ/Δ*^ mice showed the phenotypes similar to control mice in DSS-induced colitis (see Figures S4B and C).

The Wnt/β-catenin pathway in intestinal DCs is required for immunosuppression^35^. Although Wnt5a has been shown to inhibit the β-catenin pathway^1^,^4^, mRNA expression levels of *Axin2*, a target gene of β-catenin signaling, did not increase in CD11c^+^ DCs from *Wnt5a*^*CAGΔ/Δ*^ and *Ror2*^*MxΔ/Δ*^ mice (Fig. 6a,b). This suggested that the phenotypes induced by *Wnt5a* and *Ror2* deficiency were not due to the activation of the Wnt/β-catenin pathway.

### The Wnt5a-Ror2 axis was also involved in functions of bone marrow-derived DCs

**Figure 7:**
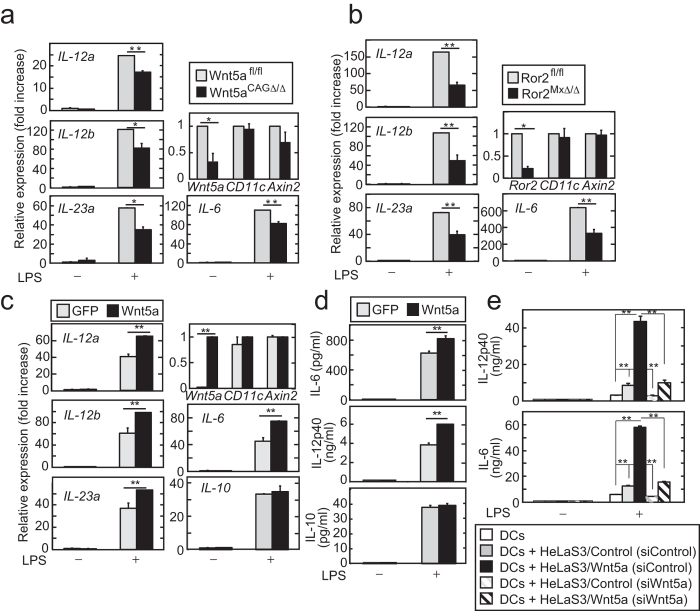
The Wnt5a-Ror2 axis is involved in pro-inflammatory cytokine synthesis in BMDCs. (**a**) BMDCs isolated from *Wnt5a*^*fl/fl*^ or *Wnt5a*^*CAGΔ/Δ*^ mice were stimulated with 100 ng/ml LPS for 4 h. The mRNA levels of the indicated cytokines, *CD11c,* and *Axin2* were measured by quantitative RT-PCR, and expressed as fold increases compared with expression levels from control BMDCs without LPS stimulation. (**b**) BMDCs from *Ror2*^*fl/fl*^ or *Ror2*^*MxΔ/Δ*^ mice were stimulated with 100 ng/ml LPS for 4 h. The mRNA levels of the indicated genes were measured by quantitative RT-PCR. (**c** and **d**) During the differentiation to BMDCs isolated from WT mice, cells were infected with lentiviruses expressing GFP or Wnt5a at day 3. BMDCs were then stimulated with 10 ng/ml LPS for 4 h (**c**) or with 10 ng/ml LPS for 8 h (**d**). The mRNA levels of the indicated genes (**c**) or the concentrations of the indicated cytokines in the culture supernatants (**d**) were measured. (**e**) BMDCs cocultured for 7 days with HeLaS3/Control or HeLaS3/Wnt5a cells transfected with indicated siRNAs were stimulated with 10 ng/ml LPS for 8 h. The concentrations of IL-6 and IL-12 p40 in the culture supernatants were measured by ELISA. Two (**a** and **b**) indicated conditional knockout mice or two WT mouse (**c**–**e**) were used in one experiment. The results are shown as means ± SE from three independent experiments. *P < 0.05, **P < 0.01 as calculated by the Student’s *t*-test.

To examine the roles of Wnt5a signaling in DC functions further, bone marrow cells were isolated from *Wnt5a*^*CAGΔ/Δ*^ and *Ror2*^*MxΔ/Δ*^ mice and incubated with granulocyte macrophage colony-stimulating factor (GM-CSF) for 7 days to generate bone marrow-derived DCs (BMDCs). Loss of *Wnt5a* or *Ror2* suppressed *IL-12a*, *IL-12b*, *IL-23a*, and *IL-6* mRNA expression in BMDCs stimulated with LPS (Fig. 7a,b). Ectopic expression of Wnt5a did not affect the basal levels of *IL-12a*, *IL-12b*, *IL-23a*, *IL-6, and IL-10* mRNA expression, but did enhance LPS-induced pro-inflammatory cytokine mRNA expression (Fig. 7c). ELISA confirmed that ectopically expressed Wnt5a promotes LPS-induced secretion of IL-6 and IL-12p40 but not that of IL-10 (Fig. 7d). However, Wnt5a did not affect the expression of CD80 and CD86, which indicate DC maturation, in BMDCs (Figure S8), suggesting that Wnt5a signaling did not affect the phenotype of DCs.

To test whether Wnt5a secreted from other cells affects DC functions, bone marrow cells were cocultured with mouse fibroblast cell lines, L cells stably expressing Wnt5a in the presence of GM-CSF for 7 days. However, this coculture experiment failed, because L cells were detached from dishes after 5 days. Instead, HeLaS3 cells stably expressing Wnt5a (HeLaS3/Wnt5a cells) were used in this experiment (Figure S9A). Knockdown of Wnt5a efficiently reduced both endogenous and exogenous Wnt5a and the phosphorylation of Dishevelled 2 (Dvl2), which indicates the activation of Wnt5a signaling (Figure S9A). HeLaS3 cells neither expressed *IL-12* mRNA nor induced *IL-6* mRNA in response to LPS regardless of Wnt5a expression levels (Figure S9B). Under these conditions, coculture of bone marrow cells with HeLaS3/Control cells promoted LPS-induced secretion of IL-12p40 and IL-6 in the resultant BMDCs (Fig. 7e), because HeLaS3 cells express Wnt5a endogenously^36^ (Figure S9A). Coculture with HeLaS3/Wnt5a cells further enhanced it (Fig. 7e). Enhanced secretions of IL-12p40 and IL-6 were suppressed by knockdown of *Wnt5a* in these HeLaS3 cells (Fig. 7e). Taken together, these results suggested that the Wnt5a-Ror2 axis between non-hematopoietic cells and DCs promoted pro-inflammatory cytokine production in DCs.

### The Wnt5a-Ror2 axis promoted the priming action of IFN-γ in DCs

Finally, the mechanism by which Wnt5a promotes inflammation was examined. LPS activates nuclear factor kappa B (NF-κB), JNK, and p38 through TLR4 to induce the expression of IL-12, IL-6, and TNF-α in DCs^37^. When control BMDCs from *Wnt5a*^*fl/fl*^ and *Ror2*^*fl/fl*^ mice were stimulated with LPS, inhibitor of kappa B-α (IκB-α) was degraded to induce the nuclear translocation of the NF-κB p65-subunit (NF-κBp65), and JNK and p38 were also phosphorylated (Figures S10A-D). There were no significant differences in the activation of these signaling pathways in BMDCs from *Wnt5a*^*CAGΔ/Δ*^ and *Ror2*^*MxΔ/Δ*^ mice compared with *Wnt5a*^*fl/fl*^ and *Ror2*^*fl/fl*^ mice, respectively (Figures S10A-D). Therefore, the Wnt5a-Ror2 axis might promote LPS-dependent cytokine production without affecting the TLR4 signaling pathway directly.

**Figure 8:**
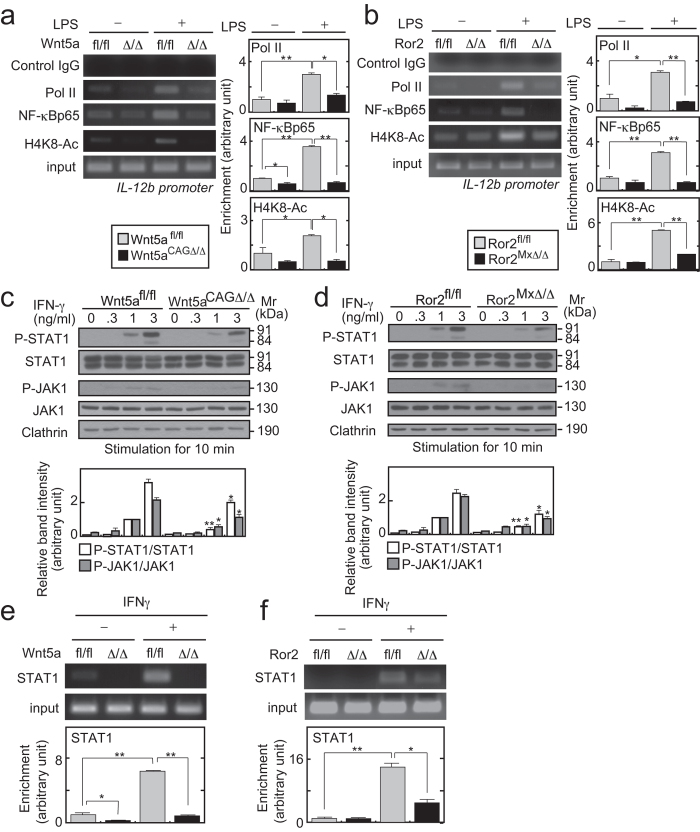
The Wnt5a-Ror2 axis enhances LPS-dependent IL-12 synthesis by promoting the priming action of IFN-γ in DCs. (**a** and **b**) BMDCs from *Wnt5a*^*fl/fl*^ *or Wnt5a*^*CAGΔ/Δ*^mice (**a**) and *Ror2*^*fl/fl*^ *or Ror2*^*MxΔ/Δ*^ mice (**b**) were stimulated with 100 ng/ml LPS for 4 h. The recruitment of RNA polymerase II (Pol II), NF-κBp65, and acetylated histone H4 lysine 8 (H4K8-Ac) to the promoters of the *IL-12b* gene was assessed by ChIP. Representative ChIP results (*left panels*). The results are expressed as arbitrary units compared with the signal intensities in control cells without LPS stimulation (*right panels*). (**c** and **d**) BMDCs from *Wnt5a*^*fl/fl*^ *or Wnt5a*^*CAGΔ/Δ*^ mice (**d**) and *Ror2*^*fl/fl*^ *or Ror2*^*MxΔ/Δ*^mice (**e**) were stimulated with the indicated concentrations of IFN-γ for 10 min. The lysates were probed with the indicated antibodies and the results shown are representative of three independent experiments (*top panels)*. The signals detected by indicated antibodies were quantified by NIH image and expressed as arbitrary units (*bottom panel*). Cropped blots are used. Full scan images of immunoblots are presented in Figures S11 and S12.(**e** and **f**) BMDCs from *Wnt5a*^*fl/fl*^ and *Wnt5a*^*CAGΔ/Δ*^ mice (**e**) and *Ror2*^*fl/fl*^ and *Ror2*^*MxΔ/Δ*^ mice (**f**) were stimulated with 30 ng/ml IFN-γ for 4 h. The recruitment of STAT1 to the promoters of the *IL-12b* gene was assessed by ChIP. Representative ChIP results (*top panels*). The results are expressed as arbitrary units compared with the signal intensities in control cells without LPS stimulation (*bottom panels*). Three indicated conditional knockout mice were used in one experiment. The results are shown as means ± SE from three independent experiments. *P < 0.05, **P < 0.01 as calculated by the Student’s *t*-test.

IL-12 from DCs and macrophages plays a critical role in Th_1_ differentiation^15^,^38^, and FACS analyses revealed that IFN-γ-producing Th_1_ cells in the colon were decreased in *Wnt5a*^*CAGΔ/Δ*^mice (see Fig. 3c,d). Therefore, the mechanism underlying how the Wnt5a-Ror2 axis affects the transcription of the *IL-12b* gene in DCs was examined using the chromatin immunoprecipitation (ChIP) assay. LPS induces the recruitment of RNA polymerase II (Pol II) and NF-κBp65 to the *IL-12b* promoter in macrophages^39^. Pol II and NF-κBp65 were recruited to the *IL-12b* promoter region by LPS stimulation in BMDCs from *Wnt5a*^*fl/fl*^ and *Ror2*^*fl/fl*^ mice, but their recruitment was diminished in BMDCs from *Wnt5a*^*CAGΔ/Δ*^ and *Ror2*^*MxΔ/Δ*^ mice (Fig. 8a,b). LPS also enhanced histone H4K8-acetylation (H4K8-Ac) at the *IL-12b* gene in BMDCs from *Wnt5a*^*fl/fl*^ and *Ror2*^*fl/fl*^ mice, which was decreased in BMDCs from *Wnt5a*^*CAGΔ/Δ*^ and *Ror2*^*MxΔ/Δ*^mice (Fig. 8a,b). Thus, the Wnt5a-Ror2 axis may enhance LPS-induced transcription of the *IL-12b* gene in DCs through the formation of Pol II-containing transcription initiation complexes.

IFN-γ primes macrophages for the expression of *IL-12b* gene^16^,^40^,^41^. Given that the actions of Wnt5a are similar to the effects of IFN-γ priming, we examined whether the Wnt5a-Ror2 axis affects IFN-γ signaling in DCs. IFN-γ induced the phosphorylation of JAK1 and STAT1 in BMDCs from *Wnt5a*^*CAGΔ/Δ*^ and *Ror2*^*MxΔ/Δ*^ mice, but at a lower level than was observed in control BMDCs from *Wnt5a*^*fl/fl*^ and *Ror2*^*fl/fl*^ mice (Fig. 8c,d). In addition, the IFN-γ-induced recruitment of STAT1 to the *IL-12b* promoter was decreased in BMDCs from *Wnt5a*^*CAGΔ/Δ*^ and *Ror2*^*MxΔ/Δ*^ mice compared with their control BMDCs (Fig. 8e,f). However, Src activity, which is involved in the Wnt5a and STAT signaling^42^,^43^, was not changed in BMDCs from *Wnt5a*^*CAGΔ/Δ*^ and *Ror2*^*MxΔ/Δ*^ mice (Figures S10E and F). Taken together, these results suggested that the Wnt5a-Ror2 axis enhances the priming action of IFN-γ, although the signaling pathway by which Wnt5a activates to regulate this action is not known at present.

## Discussion

Our results support that the Wnt5a-Ror2 axis promotes DSS-induced colitis by enhancing pro-inflammatory cytokine production in the colon. There are two major pathways for Wnt signaling. The first is the β-catenin dependent pathway, and the second is the β-catenin independent pathway, which is activated by Wnt5a. The β-catenin dependent pathway is activated in intestinal DCs and is required for the secretion of immunosuppressive cytokines^35^. Although Wnt5a signaling is able to suppress the β-catenin dependent pathway^1^,^4^, our results showed that the β-catenin dependent pathway was not activated in CD11c^+^ DCs from *Wnt5a*^*CAGΔ/Δ*^ and *Ror2*^*MxΔ/Δ*^ mice. Therefore, the β-catenin independent pathway may regulate intestinal inflammatory responses independently of the β-catenin dependent pathway at least in DCs. It is intriguing to speculate that Wnt signaling may trigger anti- or pro-immune responses through the β-catenin dependent or independent pathway, respectively, in intestinal DCs.

The clinical signs, histological damage, and pro-inflammatory cytokine levels in DSS-induced colitis were suppressed in *Wnt5a*^*CAGΔ/Δ*^ and *Ror2*^*MxΔ/Δ*^ mice compared with control mice. In turn, the *Wnt5a*^*MxΔ/Δ*^ and *Wnt5a*^*VilΔ/Δ*^ mice showed colitis phenotypes similar to control mice. The latter finding suggests that Wnt5a released from hematopoietic cells (*Wnt5a*^*MxΔ/Δ*^) or epithelial cells (*Wnt5a*^*VilΔ/Δ*^) in the colon was not involved in DSS-induced colitis. Our results demonstrated that in mice, the basal expression level of Wnt5a in intestinal fibroblasts is much higher than hematopoietic cells or epithelial cells, and that DSS administration induces Wnt5a expression in fibroblasts located in ulcer lesions. The observation that LPS-induced pro-inflammatory cytokines produced from CD11c^+^ DCs in *Wnt5a*^*CAGΔ/Δ*^ and *Ror2*^*MxΔ/Δ*^ mice were decreased but not in *Wnt5a*^*MxΔ/Δ*^ mice also support that hematopoietic cells are not the primary source of Wnt5a in the colon. In addition, our human studies show that Wnt5a was clearly observed in vimentin-positive cells, but not macrophages, in the ulcer lesions of patients with ulcerative colitis and Crohn’s diseases. Taken together, these results suggest that fibroblasts might be the primary source of Wnt5a at least in the colon during inflammatory bowel disease.

It has been reported that Wnt5a can act in a paracrine manner on neighboring cells. For example, the Wnt5a-Ror2 axis between osteoblast-lineage cells and osteoclast precursors enhances osteoclastogenesis, while Ror2 deficiency in osteoclast precursors leads to impaired osteoclastogenesis^8^. In addition, the Wnt5a-Fz8/Flamingo axis functionally maintains HSCs in their niche through interactions between N-cadherin-positive osteoblasts, which express Wnt ligands including Wnt5a, and long-term HSCs that express Fz8 and Flamingo^44^. Similarly, our results suggest that Wnt5a from non-hematopoietic cells (probably fibroblasts) act on DCs, which express Ror2, to promote inflammation in response to infectious cues. However, knockdown of *Wnt5a* in murine macrophages suppresses the secretion of IFNs *in vitro*^12^. Wnt5a secreted from human DCs is involved in IL-12 secretion in autocrine manner and enhances IFN-γ secretion from CD4^+^ T cells in paracrine manner^45^. Thus, it cannot be excluded the possibility that Wnt5a promotes cytokine production from hematopoietic cells in an autocrine manner, at least *in vitro*, in different conditions. Intestinal DCs are the first cells recruited to and activated at sites of infection or injury. DCs play an important role in the gut where they continually sample intestinal antigen and participate in inducing distinct immune responses^21^. IL-12 production from DCs in particular is essential for Th_1_ differentiation^15^,^38^, and IFN-γ-producing Th_1_ cells in the colon were indeed decreased in the *Wnt5a*^*CAGΔ/Δ*^mice in this study. Although it might be possible that the Wnt5a-Ror2 axis is involved in the recruitment of naïve T cells into the colon^22^,^23^, this possibility is unlikely, because the T cell number in the colon was not changed in *Wnt5a*^*fl/fl*^ and *Wnt5a*^*CAGΔ/Δ*^ mice (see Figure S3C). Therefore, our results provided one possible mechanistic link between Wnt5a signaling and DC functions.

Wnt5a reportedly promoted NF-κB signaling through the activation of PKC or Rac in endothelial cells and macrophages^12^,^46^, but this is not the case in DCs. In our study, LPS induced degradation of IκB-α and NF-κBp65 nuclear localization in BMDCs from *Wnt5a*^*CAG*Δ/Δ^ and *Ror2*^*MxΔ/Δ*^ mice, as well as BMDCs from control mice. Instead, the Wnt5a-Ror2 axis was required for LPS-induced IL-12 expression by enhancing Pol II recruitment, NF-κBp65 occupancy, and histone acetylation. Therefore, it is likely that the Wnt5a-Ror2 axis is involved in promoting chromatin remodeling to increase recruitment of TLR-activated transcription factors at the *IL-12b* gene. This mechanism of action resembles to that of IFN-γ. As another receptor of Wnt5a, Ryk (related to receptor tyrosine kinase) atypical tyrosine kinase receptor, is involved in Wnt5a signaling-mediated axon guidance^47^,^48^ and *Xenopus* gastrulation movement^49^, and it is expressed in immature DCs and CD4^+^ T cells^45^. To clarify whether Ryk mediates Wnt5a-dependent immune responses would be necessary for the understanding of roles of Wnt5a signaling in inflammation.

An important function of IFN-γ is to prime DCs and macrophages for synergistic transcription of pro-inflammatory cytokine genes by inflammatory factors, through STAT1 activation^15^,^16^,^38^. For instance, IFN-γ induces TLR4 dependent transcription factors and RNA polymerase II to occupy cytokine gene promoters and enhancers, thereby increasing expression of TNF-α, IL-6, and IL-12, in macrophages^16^. Our results demonstrated that IFN-γ dependent JAK activation and STAT1 phosphorylation were decreased in BMDCs from *Wnt5a*^*CAG*Δ/Δ^ and *Ror2*^*MxΔ/Δ*^ mice. It is possible that the Wnt5a-Ror2 axis promotes the IFN-γ priming action in DCs. Loss of *Wnt5a* or *Ror2* did not affect the expression of JAK1 and STAT1 (see Fig. 8c,d) and the basal levels of *IFN-*γ *receptor1/2* mRNA (data not shown). Thus, Wnt5a-induced expression of other genes might decide the threshold for IFN-γ signaling although the detailed mechanism is not known at present. The reduction in the Th_1_ differentiation in *Wnt5a*^*CAGΔ/Δ*^ mice could be due primarily to the decrease in IL-12 production from DCs. IFN-γ is mainly produced by Th_1_ cells, natural killer cells, and CD8^+^ T cells. IL-12 is important for Th_1_ differentiation and IFN-γ synthesis, and IFN-γ promotes IL-12 production in DCs. Therefore, the Wnt5a-Ror2 axis would regulate the priming action of IFN-γ and contributes to the signaling circuit between IL-12 and IFN-γ.

## Methods

### Animals

The protocols used for all animal experiments in this study were approved by the Animal Research Committee of Osaka University, Japan (No. 21–048–1). All animal experiments were carried out according to the guidelines for the care and use of experimental animals of Osaka University. Details of *Wnt5a*^*fl/fl*^ mouse line generation are described in Supplementary Methods. *Ror2*^*fl/fl*^ mice were generated as described^8^. *CAG-Cre/ERT2*^*Tg*^ and *Villin-Cre*^*Tg*^ mice were purchased from Jackson Laboratory. To treat the mice, a stock solution of tamoxifen (4-hydroxitamoxifen; Sigma-Aldrich, St. Louis, MO) in ethanol (150 mg/ml) was diluted in corn oil to 15 mg/ml. The tamoxifen suspension (0.1 ml) was administered to adult *Wnt5a*^*fl/fl*^ or *Ror2*^*fl/fl*^*;CAG-Cre/ERT2*^*Tg*^ mice 4 weeks of age and relevant control *Wnt5a*^*fl/fl*^ or *Ror2*^*fl/fl*^mice three times at two day intervals to induce the activation of the Cre-ERT2 recombinase and to remove the *floxed Wnt5a* or *Ror2* coding region^19^. Mx-Cre expression was induced by the intraperitoneal injection of 500 μg pIpC (Sigma-Aldrich) six times at two day intervals to remove the *floxed Wnt5a* or *Ror2* coding region^25^. The primers used for genotyping in this study were listed in Supplementary Table S1.

### Induction of colitis

DSS was administered to eight to nine weeks old male mice in their drinking water at a final concentration of 2.5% (w/v) to induce colitis. Animals were observed daily for weight, stool consistency, and the presence of gross blood in feces and at the anus. Detailed evaluation of colitis is described in Supplementary Methods.

### Preparation of colon extraction

After 7 days administration of DSS, 2 cm length of colon from anus was isolated, and homogenized in homogenization buffer (25 mM Tris-HCl [pH 7.5], 150 mM NaCl, 1 mM phenylmethylsulfonylfluoride, 1 μg/ml leupeptin, 1 μg/ml aprotinin) by downs homogenizer (15 strokes). After the homogenization, 1% Nonidet P-40, 1% deoxycholic acid and 0.1% sodium dodecyl sulfate (SDS) were added to the homogenates. The homogenates were sonicated 6 times for 15 sec by Ultra S homogenizer (TAITEC, Nagoya, Japan), incubated at 4 ˚C with rotation for 1 h and then centrifuged at 20,000 x *g* for 10 min. The supernatants were used in ELISA.

### Patients and tissues of inflammatory bowel diseases

Tissue samples from 20 patients who underwent surgery for Crohn’s disease and ulcerative colitis at Osaka University Hospital from 2011 to 2013 were examined. Histological specimens were fixed in 10% formalin and routinely processed for paraffin embedding. Paraffin-embedded specimens were stored in the dark room in the Department of Pathology at Osaka University Hospital at room temperature and cut into 4 *μ*m thick sections at the time of staining. The study was approved by the ethics review board of Graduate School of Medicine, Osaka University (No. 13455).

### Isolation of lamina propria DCs, bone marrow-derived DCs (BMDCs), and lymphocytes

Isolations of lamina propria DCs, BMDCs, and lymphocytes were performed as described by Ueda Y. *et al.*^50^, Kayama H. *et al.*^51^, and Kusu T. *et al.*^52^, respectively. Details of experimental procedures are described in Supplementary Methods.

### Coculture of naïve CD4^+^ T cells with lamina propria DCs

Coculture of naïve CD4^+^ T cells with lamina propria DCs were performed as described by Atarashi K. *et al.*^53^ and Kayama H. *et al.*^51^. Details of experimental procedures are described in Supplementary Methods.

### General experimental procedures

General experimental procedures are described in Supplementary Methods.

### Statistical analysis

The experiments in each figure were performed three to four times, and differences between control and experimental groups were evaluated using the Student’s *t*-test and a one-way ANOVA with a Bonferroni test for multiple group comparison. A *P* value < 0.05 was considered a significant difference.

### Others

Quantification data was calculated based on at least three blots or gels from different experiments. All the gels were run under the same experimental condition as detailed in the Supplementary Methods.

## Additional Information

**How to cite this article**: Sato, A. *et al*. The Wnt5a-Ror2 axis promotes the signaling circuit between interleukin-12 and interferon-γ in colitis. *Sci. Rep.* **5**, 10536; doi: 10.1038/srep10536 (2015).

## Supplementary Material

Supplementary Information
