# Supplementary material for: The Wnt5a-Ror2 axis promotes the signaling circuit between interleukin-12 and interferon-γ in colitis
Source: Sci Rep. 2015 Jun 1;5:10536. doi: 10.1038/srep10536 (PMC4450756; doi:10.1038/srep10536)
Supplement: Supplementary Information [file srep10536-s1.pdf]

**Supplementary Information**

# The Wnt5a-Ror2 axis promotes the signaling circuit between interleukin-12 and interferon-γ in colitis

Akira Sato, Hisako Kayama, Kensaku Shojima, Shinji Matsumoto, Hirofumi Koyama, Yasuhiro Minami, Satoshi Nojima, Eiichi Morii, Hiroaki Honda, Kiyoshi Takeda, and Akira Kikuchi

## Supplementary Methods

### *Generation of Wnt5a$^{fl/fl}$* mice

A BAC clone containing the mouse *Wnt5a* gene was purchased from the BACPAC Resource Center of Children's Hospital Oakland Research Institute (Oakland, CA). A 6.5 kb genomic fragment from the *Nhe*I site in a 5' upstream region to the *Kpn*I site in intron 1, and a 3.7 kb genomic fragment from the *Kpn*I site in intron 2 to the *Kpn*I site in intron 3 were used as the 5' and 3' arms of the targeting vector, respectively. A 1.2 kb *floxed Kpn*I fragment containing exon 2 was inserted between the two arms together with a *flip-recombinase target* (*Frt*)-flanked *neomycin resistance* (*Neo*) gene. A *diphtheria toxin-A* (*DTA*) gene was attached to the 5′ end to use for negative selection. KY1.1 ES cells (provided by Dr. Junji Takeda in Osaka University in Japan) were electroporated with the targeting vector and subjected to G418 selection as described [1]. ES cells with homologous recombination were identified by blotting *Sph*I-digested genomic DNA with a *Sph*I–*Nhe*I 5′ probe and by blotting *Nco*I-digested genomic DNA with a *Kpn*I–*Nco*I 3′ probe. Correctly targeted ES cells were injected into C57BL/6×BDF1 mouse blastocysts and chimeric males were mated with C57BL/6 females to transmit the targeted allele. *Frt*-flanked *Neo* was removed by crossing the heterozygotes with *CAG–Flpe* transgenic mice (RBRC01834, provided by RIKEN BioResource Center) to generate mice with a *floxed* allele (*Wnt5a$^{+/flox}$*). *Wnt5a$^{fl/fl}$* mice were crossed with Cre transgenic mice, and conditional *Wnt5a* knockout mice were obtained. Mice that had been backcrossed to the C57BL/6-Ly5.2 background at least seven times were used for the studies.

### *Evaluation of colitis*

The following parameters were used to evaluate the severity of DSS-induced colitis [2, 3]: (i) weight

loss (0 points- none; 1 point- 1-5 % weight loss; 2 points- 5-10 % weight loss; 3 points- 10-20 % weight loss; 4 points- more than 20 % weight loss); (ii) stool consistency/diarrhea (0 points- normal; 2 points- loose stools; 4 points- watery diarrhea); and (iii) bleeding (0 points- no bleeding; 2 points- slight bleeding; 4 points- gross bleeding). DAI was calculated as the total of these scores (the sum of weight loss, diarrhea and bleeding) divided by three.

At the indicated day after DSS administration, the animals were sacrificed by $CO_2$ overdose and rapidly dissected. The entire colon was quickly removed and gently cleared of feces. Small segments of the colon were fixed with 10% normal buffered formalin and embedded in paraffin using a spin tissue processer (ThermoFisher Scientific, Waltham, MA) for histopathology and immunohistochemical analyses. The blocks were cut into 5 μm thick sections with a paraffin microtome (Leica Corp. Microsystems AG, Glattburg, Switzerland) and mounted on an MAS-coated micro slide glass (Matsunami, Osaka, Japan). Sections were stained with hematoxylin and eosin (Sakura Finetek, Tokyo, Japan) and histological epithelial damage in a 1 cm length of the colon from the anus was observed and evaluated. Epithelial damage scores were defined as follows (see Figure S2): 0 points- intact crypts; 1 point- decreased crypt lesions; 2 points- monolayer lesions; and 3 points- ulcer lesions.

***Real-time reverse transcription–PCR***

cDNAs were synthesized from RNA samples prepared with NucleoSpin RNA (MACHEREY-NAGEL, Duren, Germany) using MuLV Reverse transcriptase (Applied Biosystems, Carlsbad, CA). cDNAs were analyzed by real-time RT–PCR using Fast SYBR (Applied Biosystems) in a StepOne Real-Time RT-PCR system (Applied Biosystems). The obtained data was normalized by the expression level of *GAPDH* except for Figure 2f. In Figure 2f, the expression levels of *Wnts*

in colon were calculated by $C_T$ values, and expressed as fold increases compared with the mRNA level of *Wnt5a* at day 0. The forward and reverse primers were listed in Supplementary Table S2.

***Enzyme-linked immunosorbent assay (ELISA)***

The concentrations of IL-6, IL-12p40, TNF-α, IL-23A, IL-17A, IL-10, and IFN-γ released from the colon, BMDCs, and $CD4^+$ T cells were measured using the DuoSet ELISA Development System (R&D Systems, Minneapolis, MN) according to the manufacturer's instructions.

***In situ hybridization***

For *in situ* hybridization, unfixed mouse colon tissues were frozen in OCT compound (Sakura Finetek, Tokyo, Japan). *Wnt5a* cDNA was cloned into the pSPT18 vector. To make the RNA probes, digoxigenin-labeled antisense or sense RNA probes were synthesized with the DIG RNA Labeling Mix Kit (Sp6/T7) (Roche Diagnostics, Basel, Switzerland). Ten μm-thick sections were hybridized with RNA probes and incubated with anti-digoxigenin AP Fab fragments (Roche Diagnostics). Specific signals were visualized with nitro blue tetrazolium chloride (NBT) and 5-Bromo-4-chloro-3-indolyl phosphate (BCIP).

***Antibodies***

All antibodies used in this study except for the flow cytometry analysis were listed in Supplementary Table S3. Antibodies used in the flow cytometry analysis were listed in the method of flow cytometry.

***Immunohistochemical analysis***

For immunofluorescence staining, the entire colon was fixed in 10% formalin and embedded in paraffin. Paraffin-embedded samples were sectioned. Antigen retrieval was conducted with a Pascal pressurized heating chamber (Dako, Glostrup, Denmark) for staining. Antigen-retrieved tissue sections were blocked in PBS containing 0.5% (w/v) Triton X-100 and 40 mg/ml BSA for 30 min and incubated with primary antibodies for 3 h at room temperature. Secondary antibodies were used in accordance with the manufacturer's protocol (Life Technologies, Carlsbad, CA). The samples were examined using an All-in-one Fluorescence Microscope BZ 9000 (Keyence, Osaka, Japan).

***Isolation of lamina propria DCs***

The entire large intestines were opened longitudinally and the fecal content removed. After the intestine was incubated with HBSS containing 5 mM EDTA for 15 min at 37˚C, the tissues were washed with PBS, and the epithelial cells and muscle layers were removed with tweezers. The remaining tissue was then cut into small pieces and incubated with RPMI 1640 containing 4% fetal bovine serum (FBS), 1 mg/ml collagenase D (Roche Diagnostics, Basel, Switzerland), 0.5 mg/ml dispase (Invitrogen, Carlsbad, CA), and 40 μg/ml DNase I (Roche Diagnostics) for 1 h at 37˚C. The digested tissues were washed with HBSS containing 5 mM EDTA and filtered through a 40-μm cell strainer. The single cell suspension obtained was incubated with CD11c micro beads (Miltenyi Biotec, Bergisch Gladbach, Germany), and the lamina propria DCs were enriched via MACS separation (Miltenyi Biotec) [4]. For sorting cells by FACS, the lamina propria cells from mouse large intestines were incubated with Pacific-blue conjugated anti-CD11b and APC conjugated anti-CD11c antibodies (BD Biosciences, San Jose, CA) after blocking the Fc receptors. DCs and macrophages were sorted using a FACS Aria (BD Biosciences). The purity of the sorted cells was routinely >95%. After MACS separation or FACS sorting, cells in the flow-through fraction were cultured in

Dulbecco's modified Eagle's medium (DMEM) supplemented with 10% FBS for 12 h and the cells attached to the dish were used in assays as intestinal fibroblasts.

***Isolation of bone marrow-derived DCs (BMDCs)***

Bone marrow (BM) cells were isolated from the femurs of mice. After separation of BM mononuclear cells, BMDCs were obtained by culturing the BM cells in 10 ng/ml recombinant mouse granulocyte macrophage-colony stimulating factor (GM-CSF) (PEPROTECH, Rocky Hill, NJ) at 37˚C for 7 days in 5% CO2. On day 3 and day 5 of culture, 5 ml of RPMI-1640 medium supplemented with 10% FBS was gently removed and replaced with fresh medium and GM-CSF [5]. For treatment with IFN-γ, BMDCs were cultured in RPMI-1640 medium supplemented with 0.1% FBS for 12 h, and then stimulated with indicated concentrations of IFN-γ.

***Isolation of lymphocytes***

To prepare single-cell suspensions from mesenteric lymph nodes (MLNs), the collected MLNs were ground between glass slides, and the cells were passed through 40-μm nylon meshes [6]. For isolation of lymphocytes from lamina propria, large intestines were opened longitudinally, and washed to remove fecal content, and then shaken in HBSS containing 5 mM EDTA for 20 min at 37°C. After treatment with HBSS containing 5 mM EDTA, intestines were washed to remove epithelial cells and fat tissue, cut into small pieces, and incubated with RPMI 1640 containing 4% FBS, 1 mg/ml collagenase D (Roche Diagnostics), 0.5 mg/ml dispase (Invitrogen), and 40 μg/ml DNase I (Roche Diagnostics) for 1 h at 37°C in a shaking water bath. The digested tissues were washed with HBSS containing 5 mM EDTA and subjected to Percoll density-gradient centrifugation. Lymphocytes from

lamina propria were collected at the interface of the Percoll gradient and washed with RPMI 1640 containing 10% FBS.

***Coculture of naïve $CD4^+$ T cells with lamina propria DCs***

$CD11c^+$ DCs isolated from colonic lamina propria were used as stimulators for syngenic naïve $CD4^+$ T cells isolated from the spleen of *Wnt5a*$^{fl/fl}$ or *Ror2*$^{fl/fl}$ mice. To prepare single-cell suspension of naïve $CD4^+$ T cells from spleens, the collected organs were ground between glass slides and the cells passed through a 40-μm nylon mesh and suspended in HBSS. Splenocytes were treated with red blood cell lysis buffer (0.15 M $NH_4Cl$, 1 mM $KHCO_3$, and 0.1 mM EDTA) for 5 min and suspended in PBS containing 2% FBS. For FACS sorting, cells were stained with anti-CD4-Percp/Cy5.5, anti-CD62L-Pecy7, anti-CD25-FITC and anti-CD44-PE antibodies. Naïve $CD4^+$ T cells were collected by being sorted as $CD4^+CD25^-CD44^-CD62L^+$ cells with a FACS Aria. 2.5 x $10^5$ naïve $CD4^+$ T cells were cocultured with 1 x $10^5$ $CD11c^+$ DCs in the presence of 1 μg/ml anti-CD3 antibody for 24 h, and IFN-γ concentrations in the culture supernatants were measured by ELISA.

***Flow cytometry***

Flow cytometry analysis was performed using FACS Canto II flow cytometer (BD Biosciences) with FlowJo software (Tree Star, Ashland, OR). Cell sorting was performed using a FACS aria (BD Biosciences). The instrumental compensation was set in each experiment using single-color, two-color, or four-color stained samples.

The following antibodies were used for flow cytometry analysis: anti-CD11c-APC (HL3), anti-CD25-FITC (7D4), anti-B220-Pacifc blue (RA3-6B2), anti-CD103-FITC (M290), anti-CD103-PE (M290), anti-IL-17A-APC (TC11-18H10), and anti-CD3e-PE/Cy7 (145-2c11)

antibodies (BD Biosciences); anti-CD11b-Pacific blue (M1/70), anti-CD44-PE (1M7), and anti-CD62L-Pecy7 (MEL14) antibodies (eBioscience, San Diego, CA); anti-CX3CR1-PE (SA011F11), anti-TCR γ/δ-FITC (GL3), anti-TCR α/β-PE (H57-597), anti-IL-10-PE (JES5-16E3), anti-IFN-γ-FITC (XMG1.2), and anti-CD4-Percp/Cy5.5 (GK1.5) antibodies (Biolegend, San Diego, CA).

***Infection using lentivirus vector***

*Wnt5a* and *EGFP* cDNAs were cloned into the pLVSIN-EF1α Neo Vector (Takara Bio Inc., Shiga, Japan), to construct lentivirus vectors. Lentiviruses were produced in X293T cells by using the Lenti-XTM Lentiviral Expression Systems (Takara Bio Inc.) in accordance with the manufacturer's instructions. BM cells isolated from the femurs of mice were cultured with 10 ng/ml GM-CSF for 3 days, and infected with the lentiviruses in the presence of 10 μg/ml polybrene, centrifuged at 1000 rpm for 1 h, and incubated for 24 h with 10 ng/ml GM-CSF. After the incubation, the medium was replaced with fresh medium, and the cells were cultured with 10 ng/ml GM-CSF for an additional 7 days and then used in assays. The media was replaced on day 3 and day 5.

***Generation of HeLaS3 cells stably expressing Wnt5a***

To generate HeLaS3 cells stably-expressing Wnt5a, HeLaS3 cells were transfected with pPGK-neo/Wnt5a and selected with 400 μg/ml G418 in Dulbecco's modified Eagle's medium (DMEM) supplemented with 10% fetal bovine serum (FBS); colonies of resistant cells were isolated.

***Coculture of bone marrow cells with HeLaS3 cells stably expressing Wnt5a***

Generation of HeLaS3 cells stably expressing neomycin resistance gene (HeLaS3/Control) or Wnt5a (HeLaS3/Wnt5a) was described above. HeLaS3/Control or HeLaS3/Wnt5a cells were seeded at the densities of 2 x $10^5$ cells per a well of 6 well plate, cultured for 24 h, and inactivated by γ-irradiation for their arrested growth. Bone marrow cells isolated from the femurs of mice were cultured on the growth-arrested HeLaS3 cells in RPMI-1640 medium supplemented with 10% FBS with 10 ng/ml GM-CSF for 7 days. The media was replaced on day 3 and day 5. After 7 days, the cells were stimulated with 10 ng/ml of LPS for 8 h. The concentrations of IL-6 and IL-12p40 released from BMDCs were measured by ELISA. For normalization of the ELISA, total RNAs were extracted from the remaining cells in each well and used for quantitative RT-PCR. Because this coculture experiment included BMDCs and growth-arrested HeLaS3 cells, the concentrations of cytokines were normalized by the ratio of mouse ubiquitin mRNA contained in total RNA. For siRNA-mediated knockdown in HeLaS3 cells, control siRNA (siControl) (target sequence: 5'-CAGTCGCGTTTGCGACTGG-3') and Wnt5a siRNA (siWnt5a) (target sequence: 5'-GTTCAGATGTCAGAAGTAT-3') were used. After 2 days since transfection of siRNA, cells were growth-arrested by γ-irradiation, and used in the coculture with bone marrow cells.

***ChIP assay***

BMDCs were stimulated with 100 ng/ml LPS (Sigma-Aldrich) or 30 ng/ml IFN-γ (eBiosience) for 4 h. Chromatin was cross-linked by 1% formaldehyde at room temperature for 10 min. The cells were scrapped off the plate after washing with PBS and centrifuged at 3,000 rpm, and then the pellet was resuspended in SDS buffer (50 mM Tris-HCl [pH 8.0], 10 mM EDTA, and 0.5% SDS). The chromatin was sonicated using a Biorupotor (COSMO BIO, Tokyo, Japan), centrifuged at 14,000 rpm to remove debris, diluted 5-fold with ChIP dilution buffer (16.7 mM Tris-HCl [pH 8.0], 167

mM NaCl, 1.2 mM EDTA, and 1.1% Triton X-100) supplemented with protease inhibitors (1 mM phenylmethylsulfonylfluoride, 1 μg/ml leupeptin, 1 μg/ml aprotinin) and phosphatase inhibitors (5 mM sodium fluoride, 1 mM sodium orthovanadate, and 10 mM β-glycerophosphate), and precleared with salmon sperm DNA/protein A-agarose beads (Millipore, Darmstadt, Germany). Diluted chromatin was immunoprecipitated at 4˚C overnight, and immune complexes were absorbed with salmon sperm DNA/protein A-agarose beads, and washed one time with high salt buffer (20 mM Tris-HCl [pH 8.1], 500 mM NaCl, 0.1% SDS, 1% Triton X-100, and 2 mM EDTA), LiCl buffer (10 mM Tris-HCl [pH 8.1], 0.25 M LiCl, 1 mM EDTA, 1% deoxycholic acid, and 1% Nonidet P-40), and three times with TE buffer (10 mM Tris-HCl [pH 8.1], and 1 mM EDTA). Immune complexes were extracted in elution buffer (1%SDS, 100 mM $NaHCO_3$) and incubated for 4 h at 65˚C to revert DNA-protein cross-links. Then the DNA was extracted by incubation in proteinase K (final concentration 50 μg/ml) buffer for 1 h at 45˚C. The purified DNA was used for PCR to assess the presence of target sequences. Primers for mouse *IL-12b* promoter were as follows: 5′-AGTATCTCTGCCTCCTTCCTT-3′ and 5′-GCAACACTGAAAACTAGTGTC-3′. We analyzed the results using the PROMO database (http://alggen.lsi.upc.es/cgi-bin/promo_v3/promo/promoinit.cgi?dirDB=TF_8.3) and observed putative STAT1 binding sequences within and around the target sequence.

***Immunoblotting***

BMDCs were lysed in NP-40 buffer (20 mM Tris-HCl [pH 8.0], 137 mM NaCl, 10% glycerol, 1% Nonidet P-40, 1 mM phenylmethylsulfonylfluoride, 1 μg/ml leupeptin, 1 μg/ml aprotinin, 5 mM sodium fluoride, 1 mM sodium orthovanadate, and 10 mM β-glycerophosphate) and then centrifuged at 20,000 x *g* for 10 min. The supernatants were probed with indicated antibodies.

***Immunocytochemistry***

Cells grown on glass coverslips were fixed for 10 min at room temperature in PBS containing 4% (w/v) paraformaldehyde and then permeabilized in PBS containing 0.2% (w/v) Triton X-100 and 2 mg/ml BSA for 10 min. The cells were incubated with primary antibodies, and viewed using a confocal microscope (LSM510, Carl-Zeiss, Jana, Germany).

## Supplemental References


1 Honda, H. *et al.* Cardiovascular anomaly, impaired actin bundling and resistance to Src-induced transformation in mice lacking p130Cas. *Nat. Genet.* **19,** 361-365 (1998).

2 Okayasu, I. *et al.* A novel method in the induction of reliable experimental acute and chronic ulcerative colitis in mice. *Gastroenterology* **98,** 694-702 (1990).

3 Cooper, H. S., Murthy, S. N., Shah, R. S. & Sedergran, D. J. Clinicopathologic study of dextran sulfate sodium experimental murine colitis. *Lab. Invest.* **69,** 238-249 (1993).

4 Ueda, Y. *et al.* Commensal microbiota induce LPS hyporesponsiveness in colonic macrophages via the production of IL-10. *Int. Immunol.* **22,** 953-962 (2010).

5 Kayama, H. *et al.* NFATc1 mediates Toll-like receptor-independent innate immune responses during *Trypanosoma cruzi* infection. *PLoS Pathog.* **5,** e1000514 (2009).

6 Kusu, T. *et al.* Ecto-nucleoside triphosphate diphosphohydrolase 7 controls Th17 cell responses through regulation of luminal ATP in the small intestine. *J Immunol* **190,** 774-783 (2013).

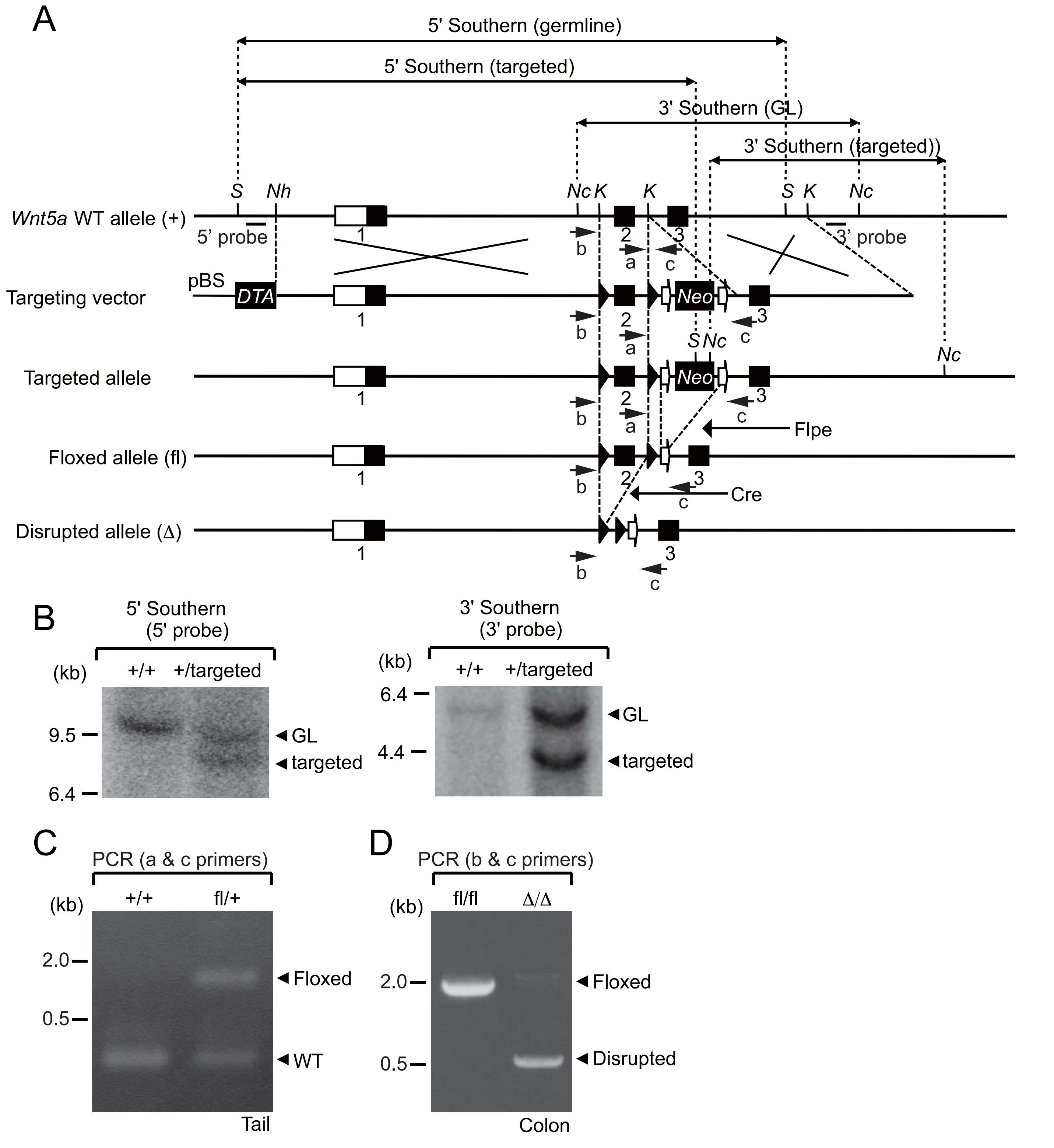


**Supplementary Figure S1. Generation of *Wnt5a*$^{fl/fl}$ mice.**

(**A**) The targeting strategy to create the *Wnt5a*-floxed allele is illustrated. The loxP sites (black arrow heads), flippase (Flpe) recognition target sites (white arrows), and the neomycin-resistance cassette (Neo), are indicated. Arrows indicate positions of primers used in PCR genotyping (a, b and c primers). The boxes with numbers indicate exons. *S*; Sph I, *Nh*; Nhe I, *K*; Kpn I, *Nc*; Nco I.

(**B**) Representative results of a genomic DNA southern blot using 5′ probe (*left panel*) and 3′ probe (*right panel*).

(**C**) Representative PCR genotyping results of tail genomic DNA using the a and c primers.

(**D**) Representative PCR genotyping results using the b and c primers of genomic DNA isolated from the colon of *Wnt5a*$^{fl/fl}$ or *Wnt5a*$^{CAGΔ/Δ}$ mice.

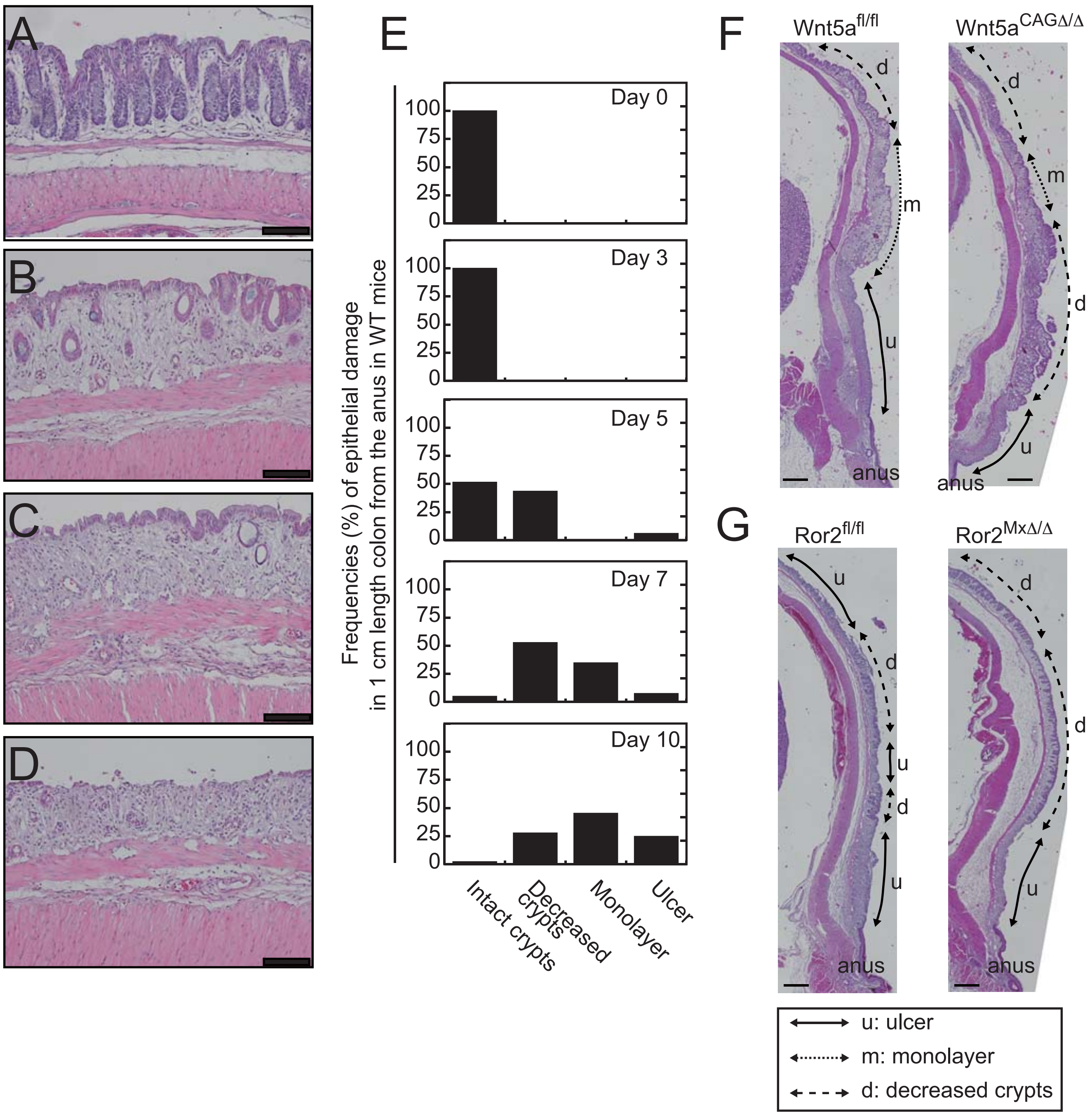


**Supplementary Figure S2. Histological analyses in DSS-induced colitis.**
(**A-D**) Hematoxylin and eosin stained tissue sections of a 1 cm section of colon from the anus at day 10 after administration of DSS. Four histological patterns were classified as follows: (**A**) intact crypts, (**B**) decreased crypt lesions, where the crypts had been partially lost, (**C**) monolayer lesions, where crypts had been completely lost but the single cell epithelial layer remained intact, and (**D**) ulcer lesions, in which the mucosal epithelial cells were disrupted. Scale bars, 100 μm.
(**E**) Histological analyses of the colon of WT mice treated with DSS for the indicated number of days were conducted, and the frequencies of four pathological patterns were expressed as percentages.
(**F** and **G**) Representative histological images of the colon isolated from *Wnt5a*$^{fl/fl}$ or *Wnt5a*$^{CAG\Delta/\Delta}$ mice (**F**) or *Ror2*$^{fl/fl}$ or *Ror2*$^{Mx\Delta/\Delta}$ mice (**G**) given DSS for 7 days. Lines with arrow heads indicates epithelium damages: d, decreased crypt lesions; m, monolayer lesions; u, ulcer lesions. Overall, ulcer lesions and decreased crypt lesions were reduced and increased, respectively, in *Wnt5a*$^{CAG\Delta/\Delta}$ and *Ror2*$^{Mx\Delta/\Delta}$ mice. Scale bars, 1 mm.

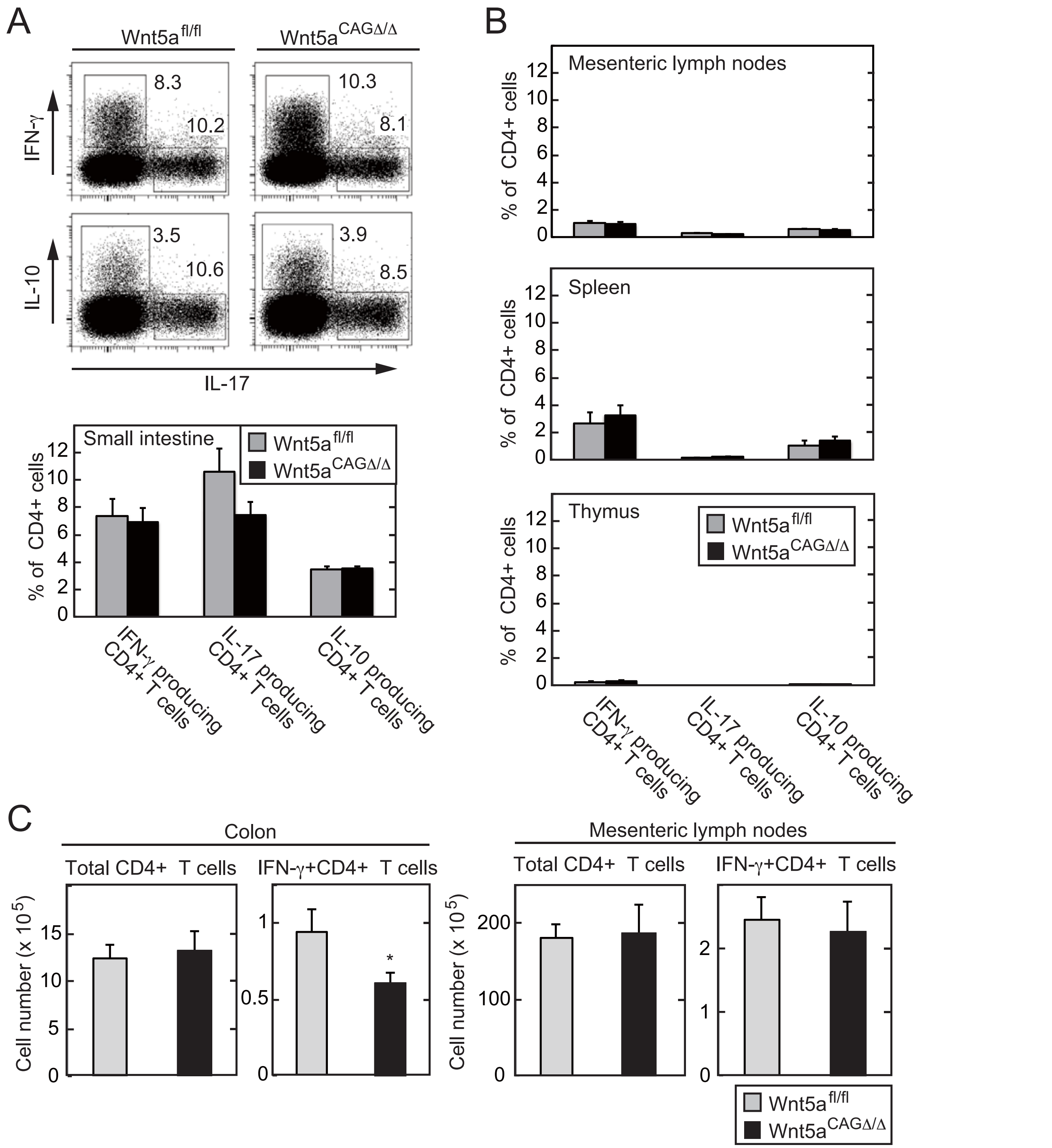


**Supplementary Figure S3. Loss of *Wnt5a* does not change the frequencies of CD4$^+$ T cells in small intestine, mesenteric lymph nodes, spleen, and thymus, and the recruitment of CD4$^+$ T cells into the colon.**
(**A**) FACS plots representing the percentage of CD4$^+$ T cells that produce IFN-γ, IL-17, and IL-10, in the small intestine from $Wnt5a^{fl/fl}$ (n= 6) and $Wnt5a^{CAG\Delta/\Delta}$ (n= 7) mice treated with water (*top panels*). The percentage of CD4$^+$ T cells that produce IFN-γ, IL-17, and IL-10 were expressed as means ± SD (*bottom panel*).
(**B**) The percentage of CD4$^+$ T cells that produce IFN-γ, IL-17, and IL-10 in the mesenteric lymph nodes (*top panel*), spleen (*middle panel*), and thymus (*bottom panel*) from $Wnt5a^{fl/fl}$ (n= 6) and $Wnt5a^{CAG\Delta/\Delta}$ (n= 7) mice treated with water were shown.
(**C**) The number of total CD4$^+$ T cells and IFN-γ$^+$CD4$^+$ T ($Th_1$) cells in the colon and the mesenteric lymph nodes isolated from $Wnt5a^{fl/fl}$ (n= 5) and $Wnt5a^{CAG\Delta/\Delta}$ (n= 5) mice was counted. The results are shown as means ± SD. *$P < 0.05$ as calculated by one-way ANOVA.

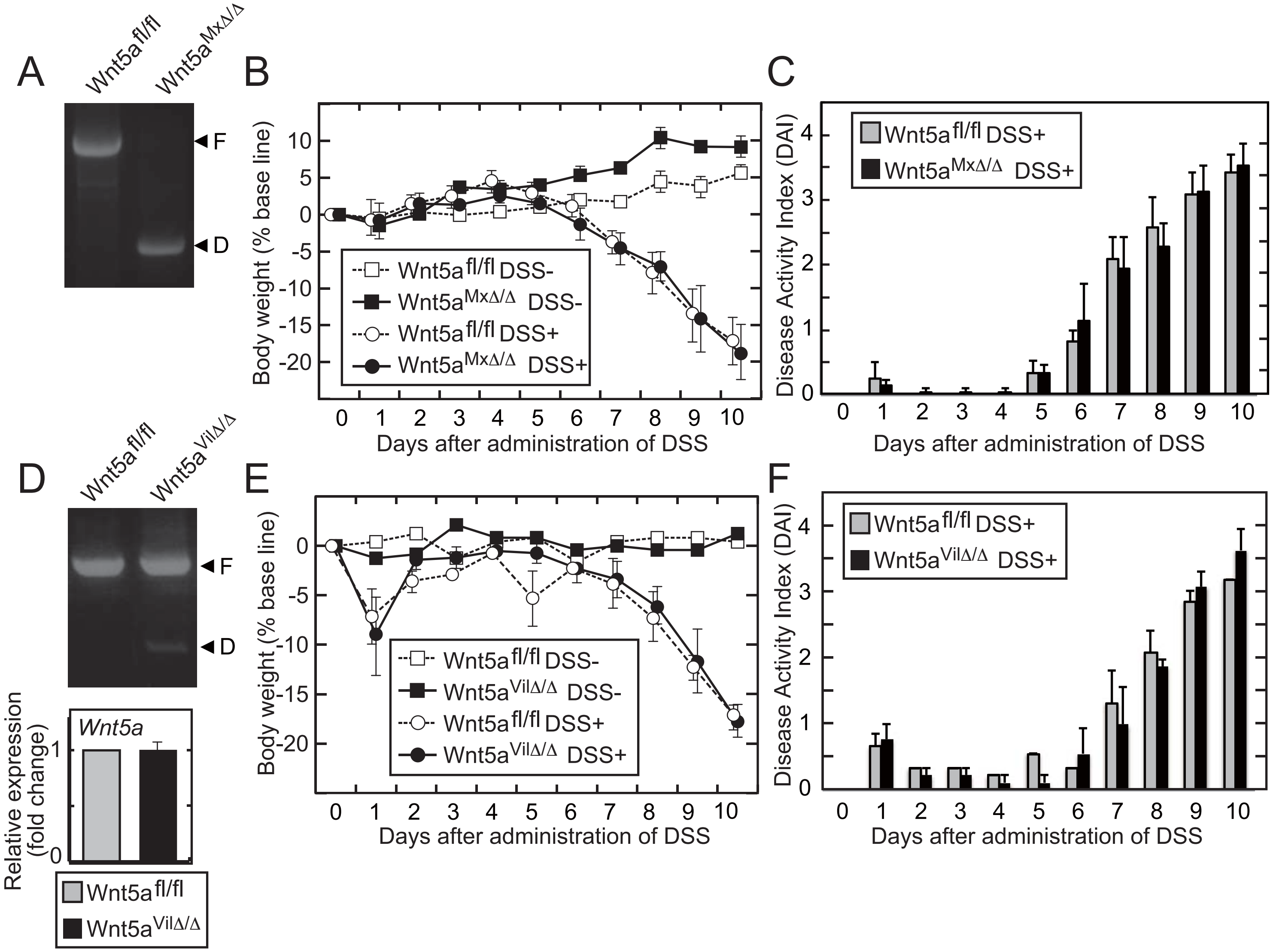


**Supplementary Figure S4. DSS-induced colitis in *Wnt5a*$^{Mx\Delta/\Delta}$ and *Wnt5a*$^{Vil\Delta/\Delta}$ mice.**

(**A**) Representative PCR genotyping results from genomic DNA extracted from the bone marrow of *Wnt5a*$^{fl/fl}$ or *Wnt5a*$^{Mx\Delta/\Delta}$ mice. F; PCR products amplified from floxed allele, D; PCR products amplified from disrupted allele.

(**B, C**) *Wnt5a*$^{fl/fl}$ (n= 4) and *Wnt5a*$^{Mx\Delta/\Delta}$ mice (n= 7) were treated with DSS for 10 days. As controls, *Wnt5a*$^{fl/fl}$ (n= 4) and *Wnt5a*$^{Mx\Delta/\Delta}$ mice (n= 7) were treated with water. The percent body weight loss of the mice (**B**) was measured daily and the DAI (**C**) was calculated.

(**D**) Representative PCR genotyping result from genomic DNA extracted from the colon of *Wnt5a*$^{fl/fl}$ or *Wnt5a*$^{Vil\Delta/\Delta}$ mice (*top panel*). The *Wnt5a* mRNA levels in the colon isolated from *Wnt5a*$^{fl/fl}$ or *Wnt5a*$^{Vil\Delta/\Delta}$ mice were measured using quantitative RT-PCR (*bottom panel*).

(**E, F**) *Wnt5a*$^{fl/fl}$ (n= 3) and *Wnt5a*$^{Vil\Delta/\Delta}$ mice (n= 3) were given DSS for 10 days. As controls, *Wnt5a*$^{fl/fl}$ (n= 3) and *Wnt5a*$^{Vil\Delta/\Delta}$ mice (n= 3) were treated with water. The percent body weight loss (**E**) and the DAI (**F**) were measured daily. The results are shown as means ± SD.

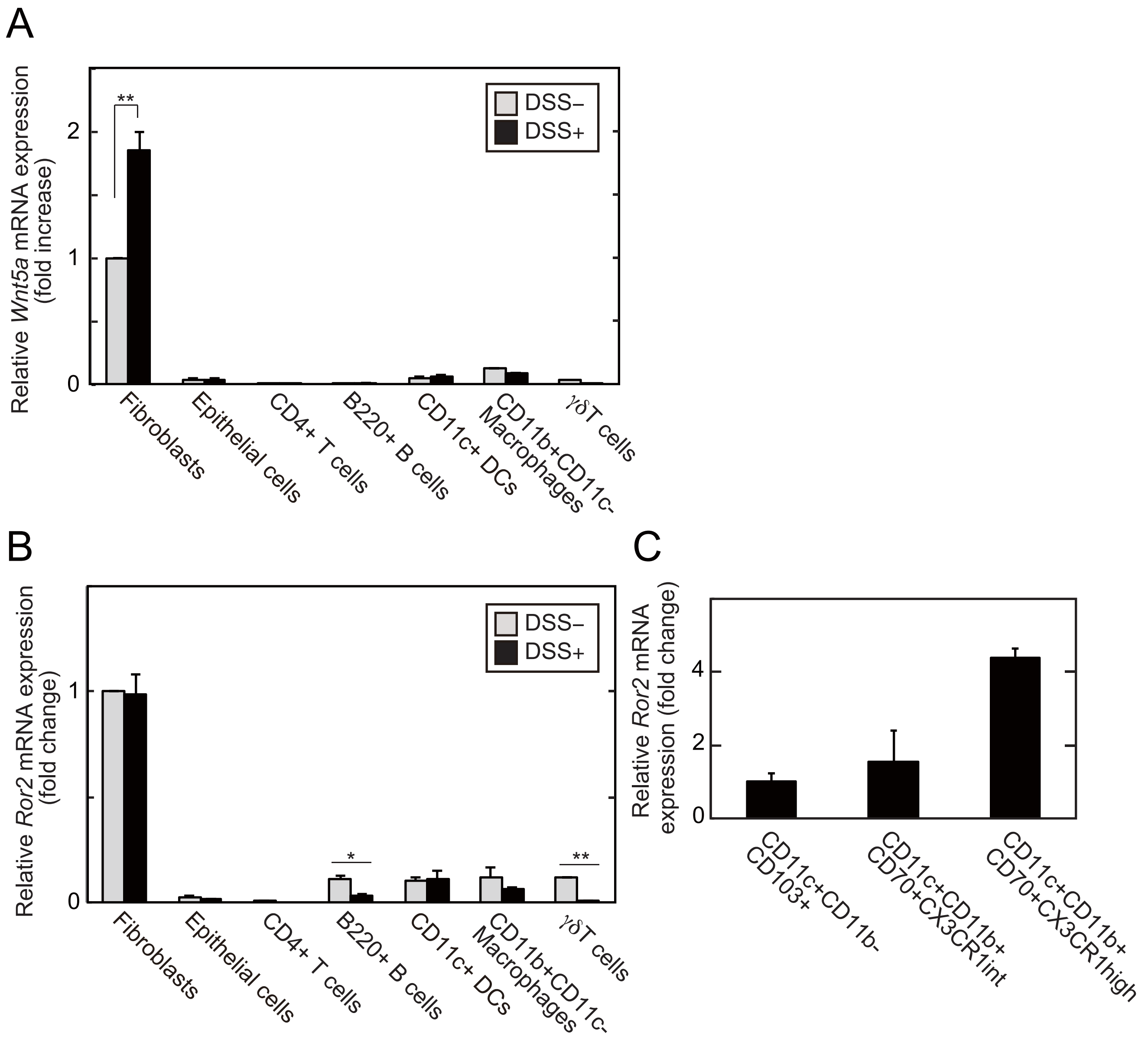


**Supplementary Figure S5. The mRNA levels of *Wnt5a* and *Ror2* in fibroblasts, epithelial cells, and hematopoietic cells in the colon.**

(**A**) Fibroblasts, epithelial cells, and hematopoietic cells including CD4$^{+}$ T cells, B220$^{+}$ B cells, CD11c$^{+}$ DCs, CD11b$^{+}$CD11c$^{-}$ macrophages, and γδT cells were isolated from the colon of *Wnt5a$^{fl/fl}$* mice given water (n= 6) or DSS (n= 6) for 8 days. The *Wnt5a* mRNA levels were measured using quantitative RT-PCR and expressed as fold increases compared with fibroblasts from water-treated mice.

(**B**) Fibroblasts, epithelial cells, and hematopoietic cells including CD4$^{+}$ T cells, B220$^{+}$ B cells, CD11c$^{+}$ DCs, CD11b$^{+}$CD11c$^{-}$ macrophages, and γδT cells were isolated from the colon of *Ror2$^{fl/fl}$* mice given water (n= 6) or DSS (n= 6) for 8 days. The *Ror2* mRNA levels were measured using quantitative RT-PCR and expressed as fold changes compared with fibroblasts from water-treated mice.

(**C**) CD11c$^{+}$ DC subsets including CD11c$^{+}$CD11b$^{-}$CD103$^{+}$ cells, CD11c$^{+}$CD11b$^{+}$CD70$^{+}$CX$_3$CR1$^{intermediate}$ cells, and CD11c$^{+}$CD11b$^{+}$CD70$^{+}$CX$_3$CR1$^{high}$ cells were isolated from the colon of WT mice given water (n= 8). The *Ror2* mRNA levels were measured using quantitative RT-PCR. Expression level of each subset was normalized to that of CD11c$^{+}$CD11b$^{-}$CD103$^{+}$ cells. The results are shown as means ± SE. *$P$ < 0.05, **$P$ < 0.01 as calculated by the Student's *t*-test.

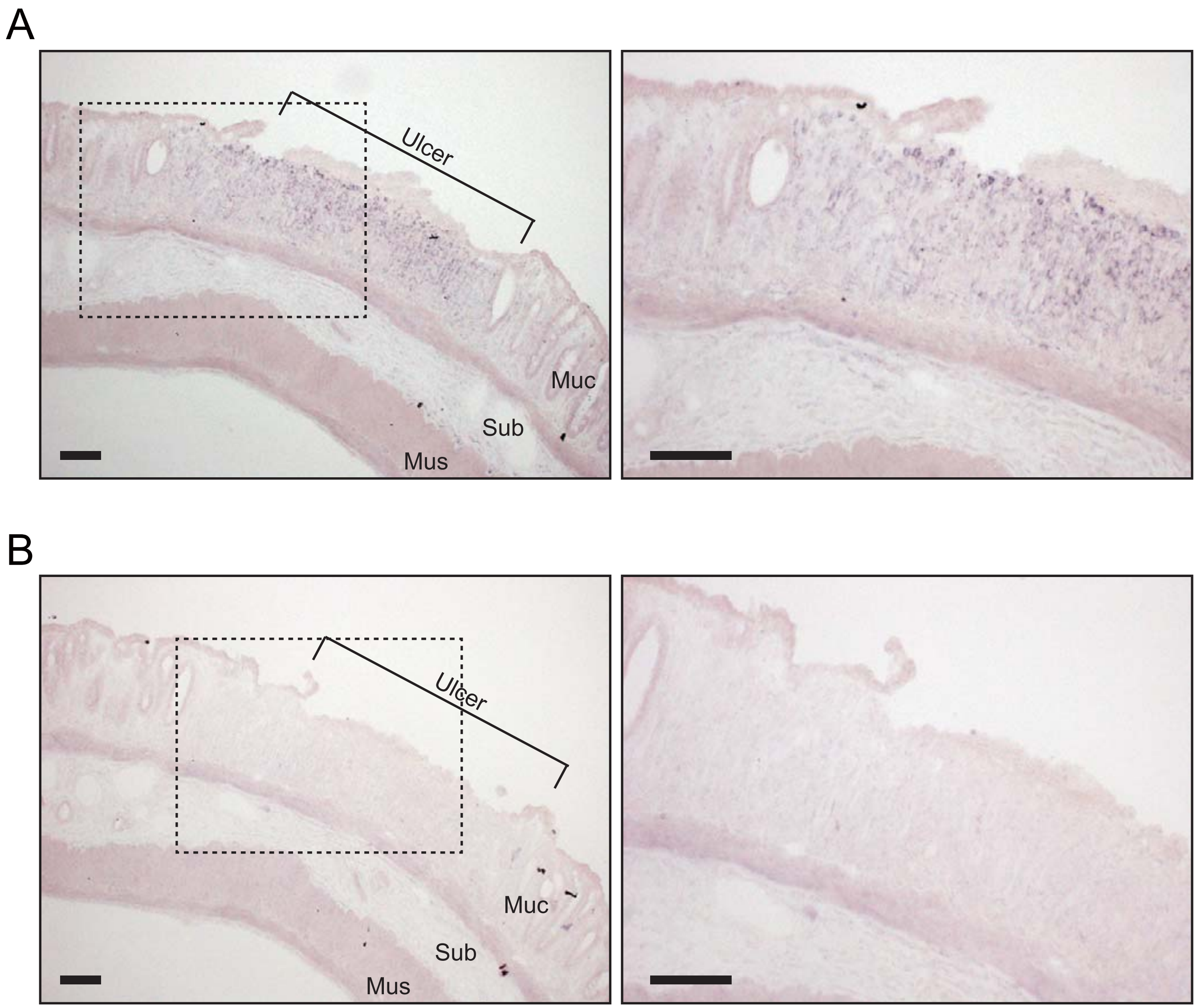


**Supplementary Figure S6. The expression patterns of *Wnt5a* mRNA in DSS-induced colitis.** (**A** and **B**) DSS was administered to WT mice for 10 days, and *Wnt5a* mRNA in the colon was detected by *in situ* hybridization using antisense probe (**A**) or control sense probe (**B**). Right panels are magnified images of a dotted rectangle in left panels. Muc; Mucosa, Sub; Submucosa, Mus; Muscularis externa. Scale bars, 100 μm.

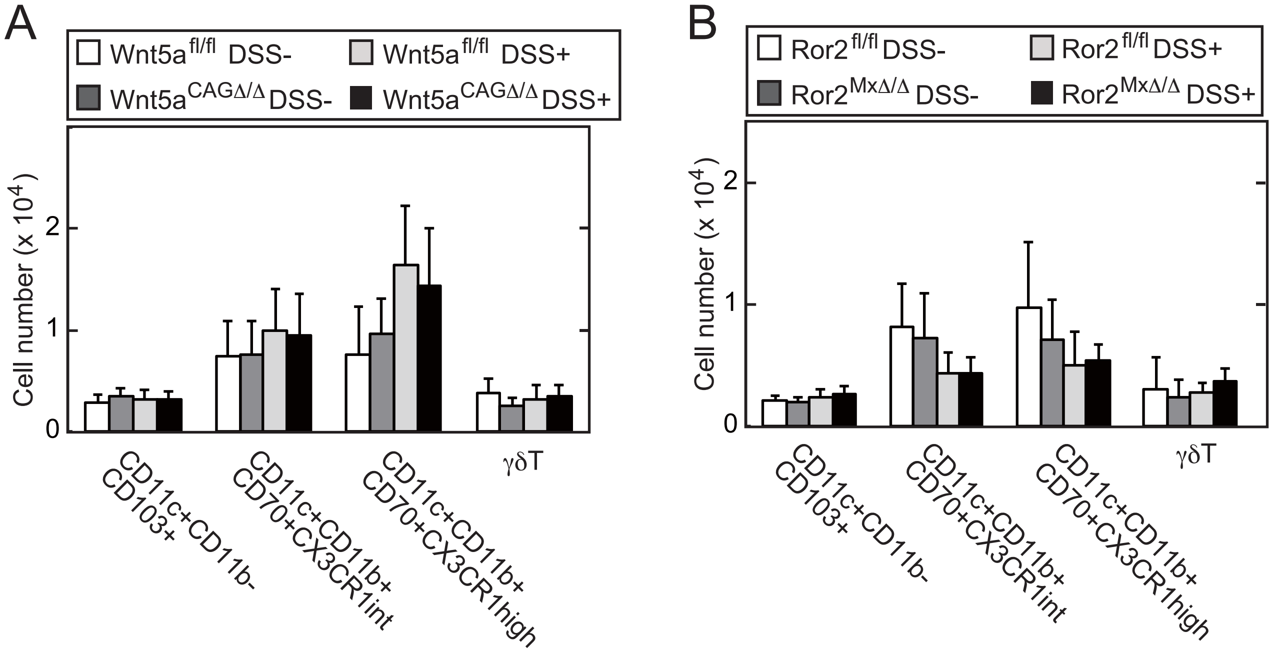


**Supplementary Figure S7. Loss of *Wnt5a* or *Ror2* does not affect cell numbers of CD11c$^+$ DC subsets and γδT cells in the colon.**

(**A** and **B**) CD11c$^+$ DC subsets, including CD11c$^+$CD11b$^-$CD103$^+$ cells, CD11c$^+$CD11b$^+$ CD70$^+$CX3CR1$^{intermediate}$ cells, and CD11c$^+$CD11b$^+$CD70$^+$CX3CR1$^{high}$ cells, and γδT cells were isolated from the colon of *Wnt5a*$^{fl/fl}$ (n=6) or *Wnt5a*$^{CAG\Delta/\Delta}$ (n=6) mice (**A**) and *Ror2*$^{fl/fl}$ (n=6) or *Ror2*$^{Mx\Delta/\Delta}$ (n=6) mice (**B**) given water or DSS for 6 days. Cell numbers of each subset were counted. The results are shown as means ± SD.

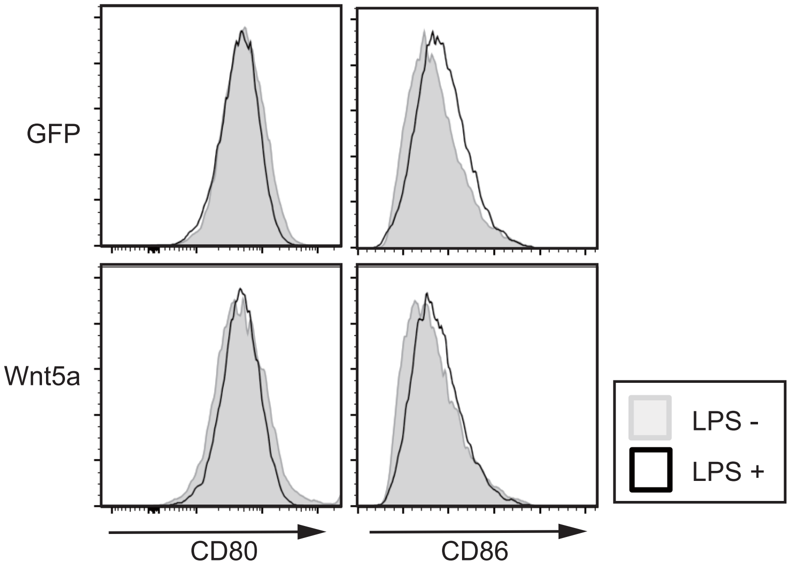


**Supplementary Figure S8. Wnt5a expression does not affect the expression of CD80 and CD86 in BMDCs.**

BMDCs infected with lentiviruses expressing GFP or Wnt5a were stained for expression of CD11c, CD80, and CD86, and CD11c$^{+}$ BMDCs were gated and then analyzed by FACS.

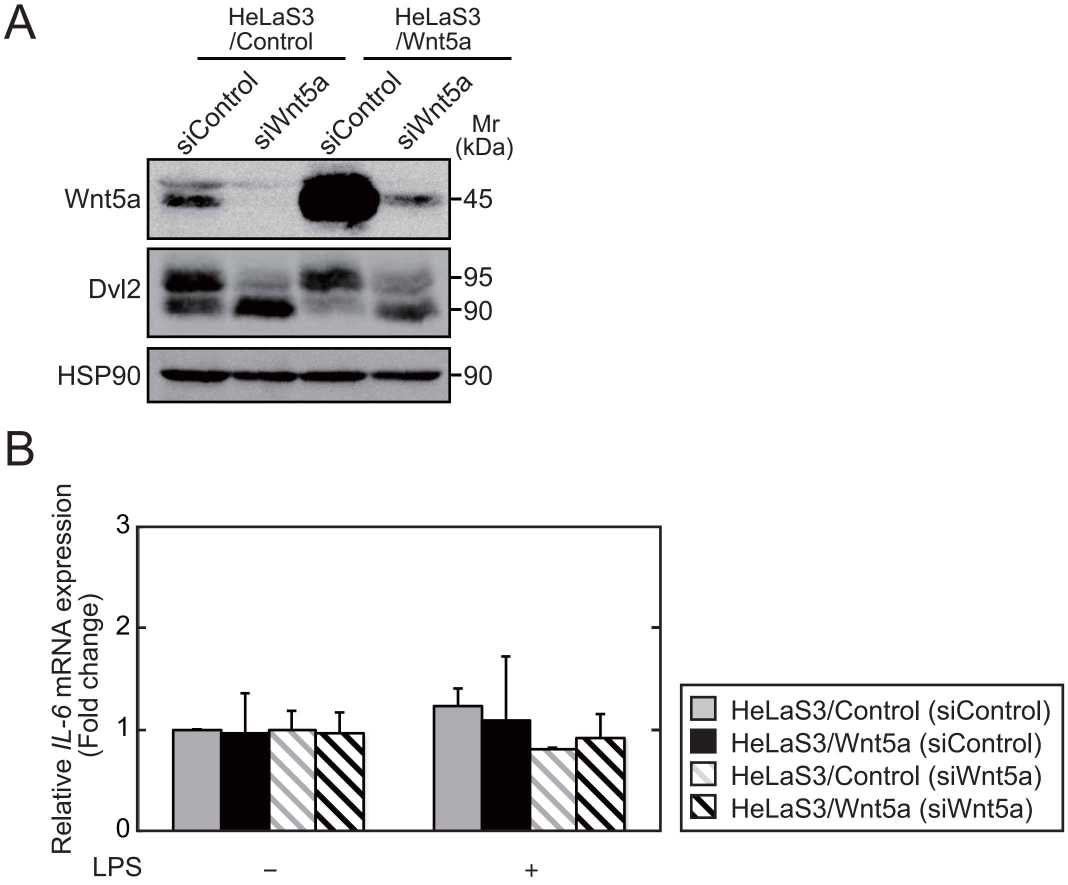


**Supplementary Figure S9. HeLaS3 cells stably expressing GFP (HeLaS3/Control cells) or Wnt5a (HeLaS3/Wnt5a cells), do not respond to LPS.**

(**A**) HeLaS3/Control or HeLaS3/Wnt5a cells were transfected with indicated siRNAs, and cultured for 3 days. The lysates were probed with indicated antibodies. The results shown are representative of three independent experiments.

(**B**) HeLaS3/Control or HeLaS3/Wnt5a cells were transfected with indicated siRNAs. After 3 days, these cells were stimulated with 10 ng/ml LPS for 4 h, and the mRNA levels of human *IL-6* were measured using quantitative RT-PCR and expressed as fold changes compared with that of HeLaS3/Control cells transfected with Control siRNA without LPS stimulation. The results are shown as means ± SE from three independent experiments.

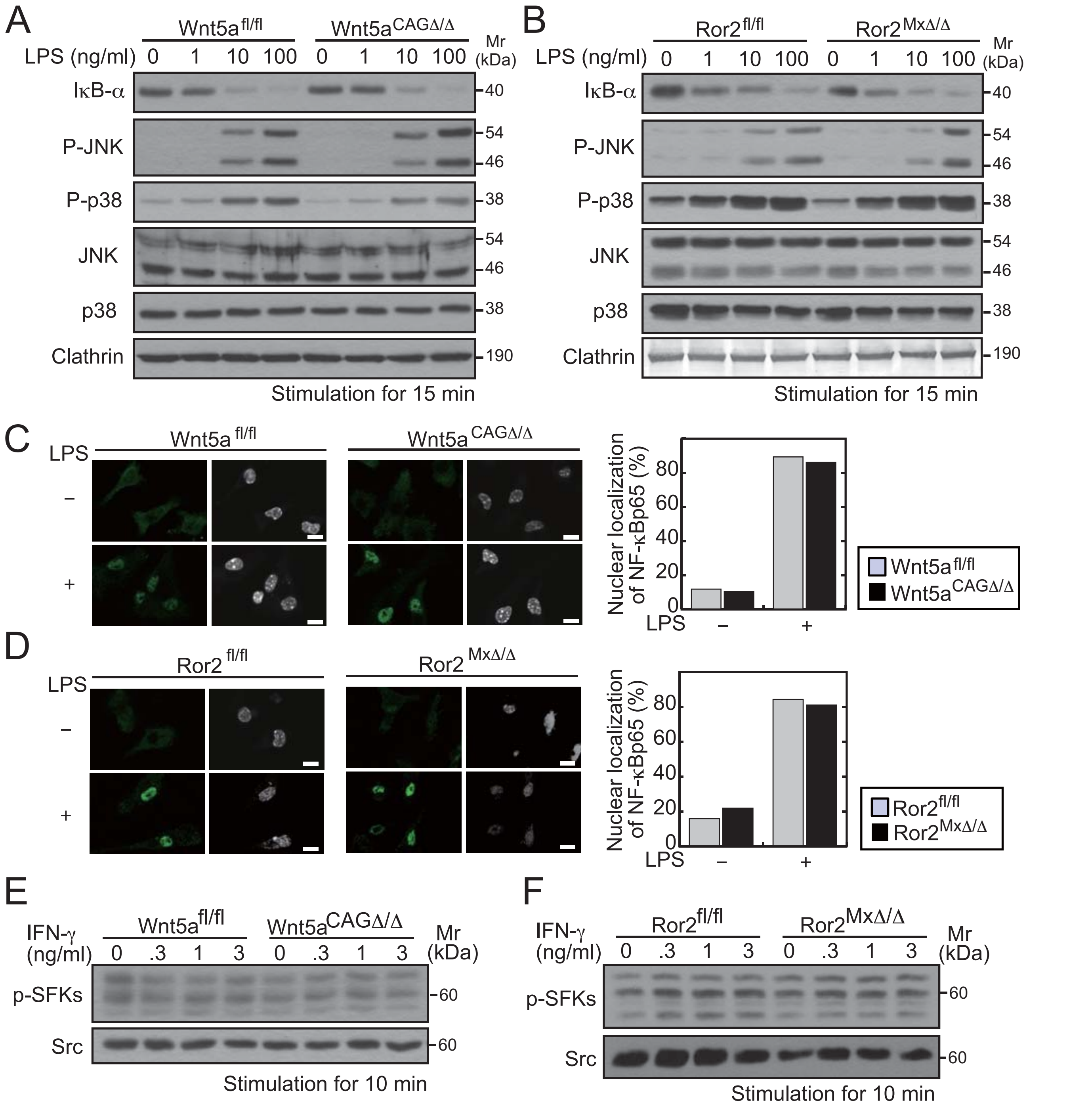


**Supplemental Figure S10. The Wnt5a-Ror2 axis dose not affect TLR4 signaling and the activation of Src family kinases (SFKs).**

**(A** and **B)** BMDCs derived from bone marrow cells isolated from *Wnt5a*$^{fl/fl}$ or *Wnt5a*$^{CAG\Delta/\Delta}$ mice (**A**) and *Ror2*$^{fl/fl}$ or *Ror2*$^{Mx\Delta/\Delta}$ mice (**B**) were stimulated with the indicated concentrations of LPS for 15 min. The lysates were probed with the indicated antibodies. The results shown are representative of three independent experiments.

**(C** and **D)** BMDCs from *Wnt5a*$^{fl/fl}$ or *Wnt5a*$^{CAG\Delta/\Delta}$ mice (**C**) and *Ror2*$^{fl/fl}$ or *Ror2*$^{Mx\Delta/\Delta}$ mice (**D**) were stimulated with 100 ng/ml LPS for 1 h, and stained with anti-NF-κBp65 antibody (green) and TO-PRO-3 (white). Representative localizations of NF-κBp65 in BMDCs with or without LPS stimulation (*left panels)*. Scale bars, 10 μm. The percentage of nuclear localization of NF-κBp65 with or without LPS stimulation (*right panels)*.

**(E** and **F)** BMDCs derived from bone marrow cells isolated from *Wnt5a*$^{fl/fl}$ or *Wnt5a*$^{CAG\Delta/\Delta}$ mice (**E**) and *Ror2*$^{fl/fl}$ or *Ror2*$^{Mx\Delta/\Delta}$ mice (**F**) were stimulated with the indicated concentrations of IFN-γ for 10 min. The lysates were probed with the indicated antibodies.

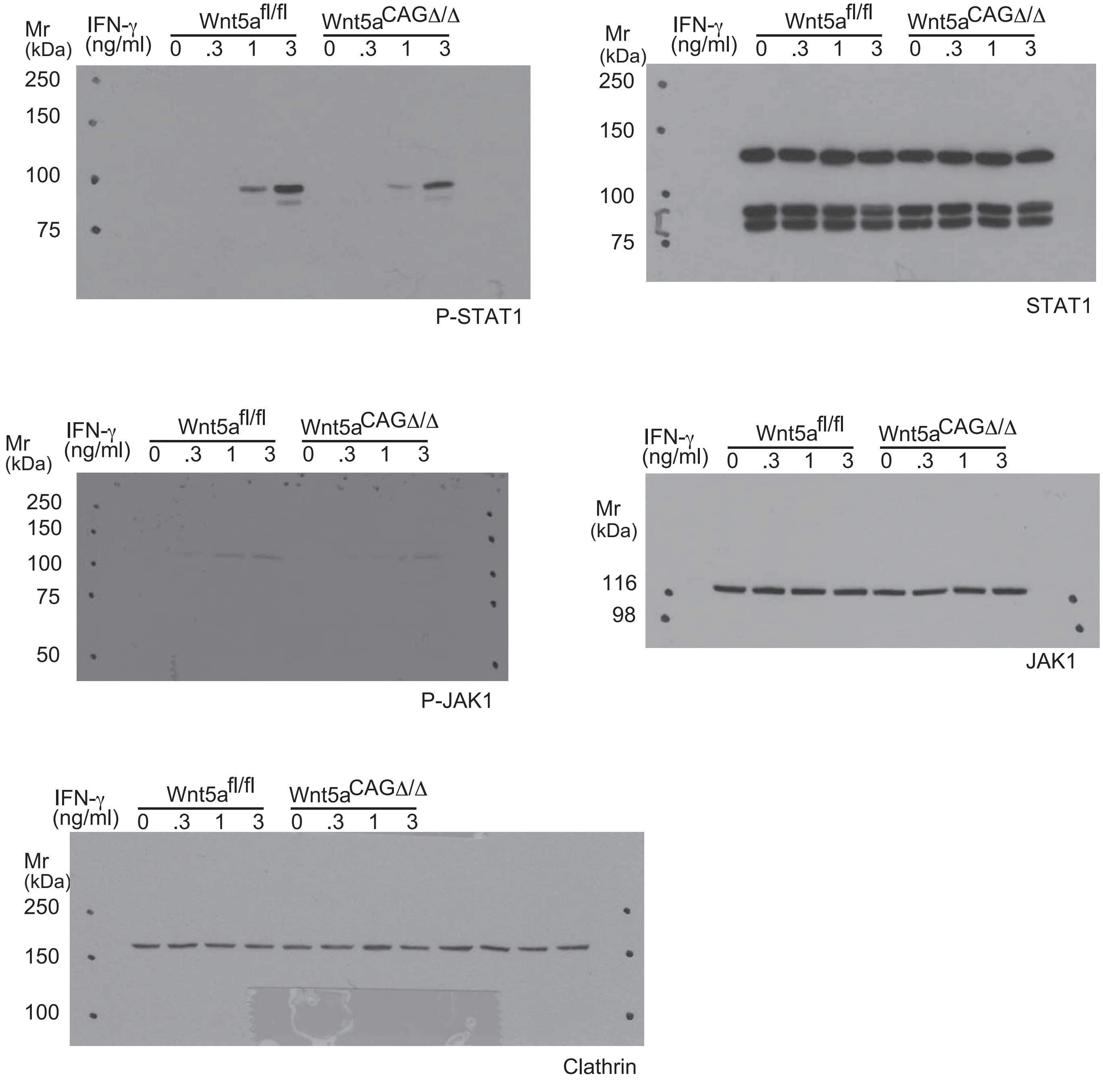


**Supplementary Figure S11. Full scan images of immunoblots presented in Figure 8c.**

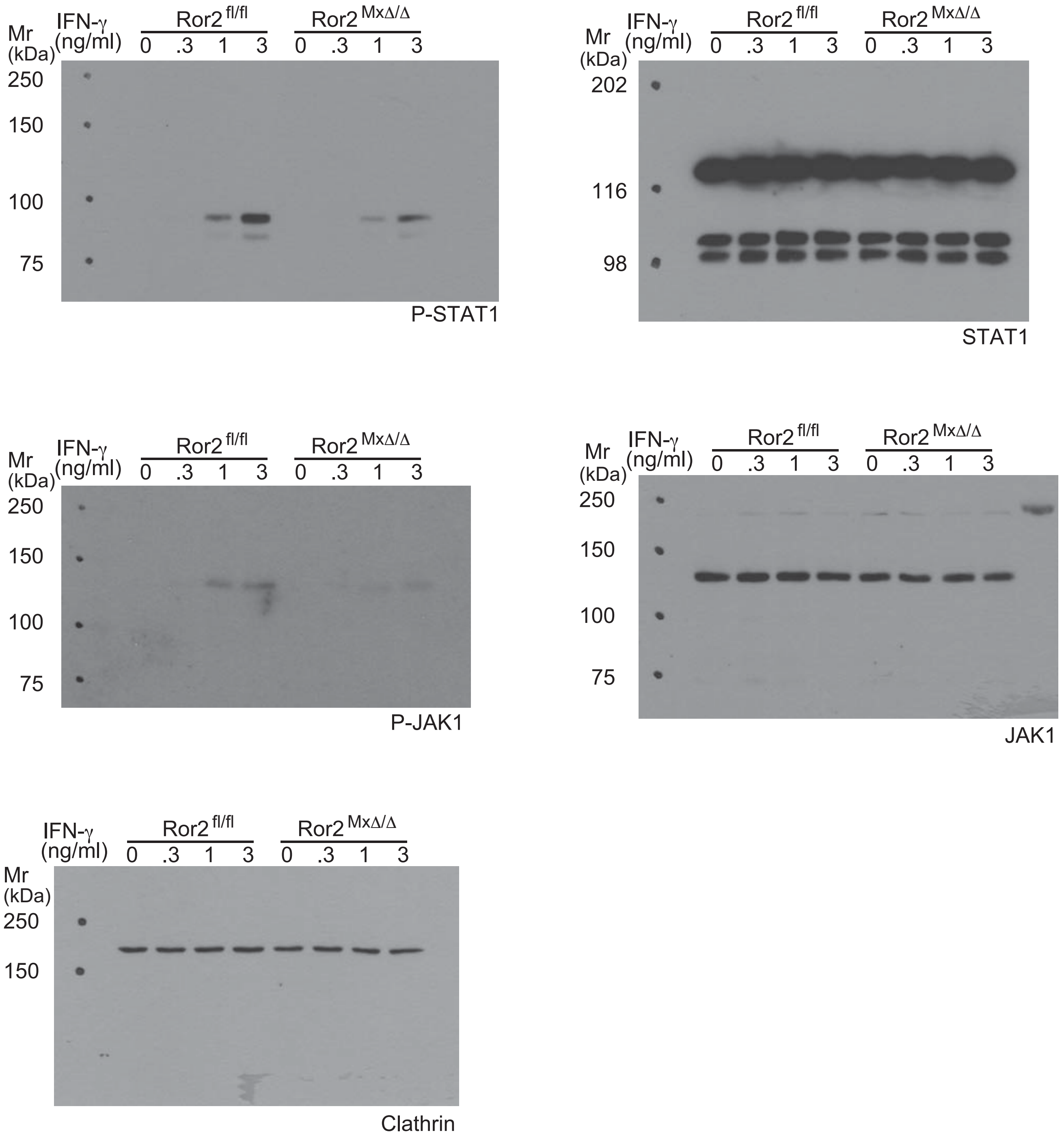


**Supplementary Figure S12. Full scan images of immunoblots presented in Figure 8d.**

**Supplementary Table S1. Forward and reverse primers for genotyping PCR used in this study.**

| Primer | Sequence |
|---|---|
| *Wnt5a wild-type allele* | ATTAAGCCCGGGAGTGGCTTT<br>GCTTCTATAACAACCTGGGCG |
| *Wnt5a conventional knockout allele* | ATTAAGCCCGGGAGTGGCTTT<br>TGGCTACCCGTGATATTGCT |
| *Wnt5a floxed allele* (fl) | ATTTCTCGGAAGGTACTGCTATCTCCTACC<br>GAAAACAGAGGTATTGTGCAGTCCCCACCC |
| *Wnt5a disrupted allele* (Δ) | GCCTTCGCGCTCTTCTCGCCCATGGAATTA<br>GAAAACAGAGGTATTGTGCAGTCCCCACCC |
| *Ror2 wild-type allele* | CTGATGTTCATCCACATACATGTGGTG<br>CCTACTATAGACTCTGATCCTTCTGCC |
| *Ror2 conventional knockout allele* | CTGATGTTCATCCACATACATGTGGTG<br>ATCGCCTTCTATCGCCTTCTTGACGAG |
| *Ror2 floxed allele* (fl) | CTCTTGTGTCAGTATGCAGG<br>GGTGGCTGAAAGCAATTAACATC |
| *Cre transgenic allele* | TGCATTACCGGTCGATGCAACGAGT<br>GAGACGGAAATCCATCGCTCGACCA |

**Supplementary Table S2. Forward and reverse primers for quantitative RT-PCR used in this study.**

| Primer | Sequence |
|---|---|
| *Wnt5a* | ATTAAGCCCGGGAGTGGCTTT<br>CTCCAATGTACTGCATGTG |
| *Wnt1* | TGGGAACTCGCATCAC<br>CTCTTGGAATCCGTCAAC |
| *Wnt3a* | CCGTCACAACAATGAGG<br>ACTCTCGGTGTTTCTCTAC |
| *Wnt4* | AGGATGCTCGGACAAC<br>GTGACACTTGCACTCCA |
| *Wnt5b* | GGTGGTCACTAGCTCT<br>CTGTCGAAACTGGTGTTG |
| *Wnt6* | TTCGGGGATGAGAAGTCAAG<br>AAAGCCCATGGCACTTACAC |
| *Wnt7a* | CAATAACGAGGCGGGT<br>CCGCTTGTTTCGACTG |
| *Wnt11* | CGTGTGCTATGGCATCAAGT<br>CATGAGCTCCAGGTTGCTG |
| *Ror2* | TCCAAGACCTGGACACAACA<br>GCCATCTTCCTGATCGTCAT |
| *IL-12a* | TACTAGAGAGACTTCTTCCACAACAAGAG<br>TCTGGTACATCTTCAAGTCCTCATAGA |
| *IL-12b* | GGAAGCACGGCAGCAGAATA<br>AACTTGAGGGAGAAGTAGGAATGG |
| *IL-23a* | GCCTGCTCTACTCCCTGATAG<br>ACTGCTGACTAGAACTCAGGC |
| *IL-6* | CCGGAGAGGAGACTTCACAG<br>TCCACGATTTCCCAGAGAAC |
| *TNF-α* | GGACAGTGACCTGGACTGTGG<br>AGTGAATTCGGAAAGCCCATT |
| *IL-17a* | TCCAGAAGGCCCTCAGACTA<br>AGCATCTTCTCGACCCTGAA |
| *IL-10* | CCAAGCCTTATCGGAAATGA<br>TTTTCACAGGGGAGAAATCG |
| *IFN-γ* | GCGTCATTGAATCACACCTG<br>TGAGCTCATTGAATGCTTGG |
| *TGF-β1* | TTGCTTCAGCTCCACAGAGA<br>TGGTTGTAGAGGGCAAGGAC |
| *CD11c* | CTGGATAGCCTTTCTTCTGCTG<br>GCACACTGTGTCCGAACTC |
| *Axin2* | CTGGCTCCAGAAGATCACAAAG |

| | |
|---|---|
| | CATCCTCCCAGATCTCCTCAAA |
| *Ubiquitin* | CGGTCTTTCTGTGAGGGTGT<br>TCACTGGGCTCCACCTCTA |
| *GAPDH* | AGCCCAGAACATCATCCCTG<br>CACCACCTTCTTGATGTCATC |
| *human IL-6* | TACCCCCAGGAGAAGATTCC<br>TTTTCTGCCAGTGCCTCTTT |
| *human IL-12b* | AAGGAGGCGAGGTTCTAAGC<br>GCAGGTGAAACGTCCAGAAT |
| *human GAPDH* | CCTGTTCGACAGTCAGCCG<br>CGACCAAATCCGTTGACTCC |

**Supplementary Table S3. Antibodies used in this study.**

| **Primary antibodies** | **Application(s)** |
|---|---|
| anti-IκBα (sc-371, SantaCruz Biotechnology) | IB |
| anti-NF-κBp65 (sc-109, SantaCruz Biotechnology) | IB |
| anti-STAT1 (#9172, Cell Signaling Technology) | IB |
| anti-phospho-STAT1 (Tyr701) (#7649, Cell Signaling Technology) | IB |
| anti-JAK1 (#3344, Cell Signaling Technology) | IB |
| anti-phospho-JAK1 (Tyr1022/1023) (#3331, Cell Signaling Technology) | IB |
| anti-SAPK/JNK (#9252, Cell Signaling Technology) | IB |
| anti-phopho-SAPK/JNK (Thr183/Tyr185) (#9251, Cell Signaling Technology) | IB |
| anti-p38MAPK (#9212, Cell Signaling Technology) | IB |
| anti-phospho-p38MAPK (Thr180/Tyr182) (#4511, Cell Signaling Technology) | IB |
| anti-phospho-Src family (Tyr416) (#6943, Cell Signaling Technology) | IB |
| anti-Src (#2123, Cell Signaling Technology) | IB |
| anti-Dvl2 (#3216, Cell Signaling Technology) | IB |
| anti-clathrin (BD Bioscience) | IB |
| anti-HSP90 (BD Bioscience) | IB |
| anti-Wnt5a/b (#2530, Cell Signaling Technology) | IB, IHC |
| anti-NF-κBp65 (sc-372, SantaCruz Biotechnology) | ICC, ChIP |
| anti-E-cadherin (610181, BD Bioscience) | IHC |
| anti-Vimentin (clone V9, Sigma-Aldrich) | IHC for human samples |
| anti-Vimentin (ab24525, Abcam) | IHC for mouse samples |
| anti-CD68 (clone: PG-M1, DAKO) | IHC for human samles |
| anti-F4/80 (clone Cl:A3-1, Abcam) | IHC for mouse samples |
| anti-CD11c (clone: N418, Abcam) | IHC for mouse samples |
| control rabbit IgG (Diagnode) | ChIP |
| anti-Pol II (sc-899, SantaCruz Biotechnology) | ChIP |
| anti-STAT1p84/p91 (sc-346, SantaCruz Biotechnology) | ChIP |
| anti-Histone H4 (acetyl K8) (ab15823, Abcam) | ChIP |

IB; immunoblotting, ICC; immunocytochemistry, IHC; immunohistochemistry, ChIP; chromatin immunoprecipitation assay.
